# Capture of long‐missing heme oxygenases that degrade both heme *b* and heme *c*

**DOI:** 10.1002/mlf2.70118

**Published:** 2026-10-28

**Authors:** Jiyuan Hou, Wenhan Dong, Hui Yang, Yuxuan Liang, Haichun Gao

**Affiliations:** ^1^ Institute of Microbiology, College of Life Sciences Zhejiang University Hangzhou China; ^2^ State Key Laboratory for Vegetation Structure Function and Construction (VegLab), College of Life Sciences Zhejiang University Hangzhou China

**Keywords:** cytochrome *c* recycling, DegQ, heme oxygenase, substrate specificity

## Abstract

Heme serves as a crucial cofactor for hemoproteins and as an iron reservoir; yet, its accumulation is toxic, necessitating efficient degradation pathways. While the catabolism of free heme *b* is well characterized, the enzymes that disrupt its counterpart covalently tethered to a peptide (heme *c*) have remained elusive. Here, through a pipeline of multiple *in silico* analyses, we predict a distinct Core‐only type of heme oxygenases (HOs) within the HugZ superfamily to have the potential to degrade both heme *b* and peptide‐tethered heme *c*. Biochemical and physiological characterization confirms that representative Core‐only‐type HOs confer balanced dual‐substrate specificity. In contrast, the C‐extension and N‐fusion‐type HOs degrade heme *b,* but not heme *c,* because the entry of heme *c* into the active pocket is blocked by their extra domains. Moreover, we show that the degradation of heme *c* by the Core‐only‐type HOs requires proteolytic digestion of heme *c*‐carrying proteins by a protease such as DegQ, which occurs in the periplasm. Overall, this work provides an integrated evolutionary, structural, biochemical, and physiological framework that defines a novel strategy for heme recycling and adaptive metabolic flexibility in bacteria.

## Impact statement

Heme oxygenases (HOs) catalyze heme degradation, playing a crucial role in maintaining heme homeostasis. Yet, HOs that work with both heme *b* and heme *c* have remained elusive. Here, we identify such enzymes, which belong to the HugZ superfamily and are universally distributed. Unlike single‐substrate HOs, these enzymes have a streamlined architecture consisting solely of the catalytic core. When coupled with a partner protease, these HOs ensure efficient recycling of heme *c* and maintain vital cellular metabolic balance.

## INTRODUCTION

Heme, an iron‐containing porphyrin molecule that serves primarily as an essential cofactor for hemoproteins, plays irreplaceable roles in a myriad of biological processes, such as oxygen transport, electron transfer, respiration, photosynthesis, oxidative stress response, signal sensing and transduction, and catalysis, in living organisms^1^, ^2^. However, when in excess, owing to its hydrophobic and lipophilic properties, heme not only readily accumulates in cellular membranes, disrupting membrane integrity, but also induces oxidative stress in host cells, damaging membranes, proteins, and DNA, leading to inflammation and tissue injury^3^, ^4^. To maintain heme homeostasis, a variety of efficient heme degradation enzymes, termed heme oxygenases (HOs), have evolved to degrade heme and recycle iron^5^, ^6^.

In cells, the initial product of all heme biosynthesis pathways known to date is heme *b*, which is not only the predominant heme species but also serves as the precursor for the generation of various derivatives, such as *a*, *d*, and *o* ^1^. In hemoproteins using these heme species as a cofactor, the association is reversible. When heme *b* is covalently attached to the conserved heme‐binding motif (HBM, typically CXXCH) of a polypeptide through thioether bonds^7^, it becomes heme *c* and the resultant hemoprotein is called cytochrome *c* (cyt *c*), which commonly plays a critical role in extracellular electron transport and/or oxidoreduction^8^, ^9^. The heme attachment of cyt *c* is a form of posttranslational modification, called cyt *c* maturation (CCM) or biosynthesis, relying on one of three dedicated protein systems, depending on the species^8^. Unlike eukaryotes, which carry at most a few cyts *c*, many bacteria encode an exceptionally large repertoire of such proteins, for example, 111 and 42 for *Geobacter sulfurreducens* ^10^ and *Shewanella oneidensis* ^11^. Moreover, a large portion of these proteins carry multiple hemes, making cyts *c* collectively a valuable pool of heme and iron that must be efficiently recycled^12^, ^13^.

Conceivably, biological systems for recycling heme *c* must have evolved. However, most HOs studied to date degrade heme *b*, whereas those shown to degrade heme *c* specifically are scarce^1^. Rat microsomal HO‐1 was reported in 1982 to possess catalytic activity against both heme *b* and heme *c*, but further validation of this function, especially *in vivo*, is still lacking^14^, ^15^. Similarly, HupZ from Group A *Streptococcus* (GAS) can moderately degrade both heme *b* and heme *c in vitro*; yet, heme *c* is unable to serve as an effective iron source for GAS *in vivo* ^16^, ^17^. Dri1 from *Synechocystis* sp. was demonstrated to show specific activity for heme *c*, but recent structural evidence suggests that the protein is a heme‐binding protein involved in metabolic sensing^18^, ^19^. The only substantiated case is *c*HOA from *Paracoccus denitrificans* (*PdcHOA*), which was shown to possess specific degradation activity toward heme *c*, but not heme *b*, without revealing its physiological significance^20^.

To date, HOs that are able to degrade both heme *b* and heme *c* remain elusive. In this study, we intended to identify the long‐missing enzymes with an *in silico* screening pipeline, including a global phylogenomic survey, reconstruction of the HO superfamily, and comparative structural analysis. We succeeded in capturing such HOs, which belong to a unique Core‐only type in the HugZ superfamily that structurally bridges the functional dichotomy between canonical heme *b* and heme *c* degradation enzymes. By combining molecular dynamics (MD) simulations and extensive site‐directed mutagenesis of representatives of these HOs, HmuZ of *S. oneidensis* and HutZ of *Vibrio cholerae*, we elucidated the molecular determinants underlying the balanced dual‐substrate capability. Furthermore, we established a DegQ‐coupled “deconstruction‐first” mechanism that overcomes the steric barrier of covalent heme attachment. These findings provide insights into the structural and biochemical plasticity of these HOs, which ensures metabolic resilience and iron homeostasis under fluctuating environmental pressures.

## RESULTS

### Global analysis of active site architectures delineates the potential substrate scope of heme oxygenases

As a first step toward identifying HOs that work with both heme *b* and heme *c*, we analyzed the organization patterns of representative bacterial genes covering all six types of HOs known to date, including HO1/HmuO, IsdG/MhuD, HugZ, PigA/HemO, ChuW, and HemS. Notably, all of these genes cluster with some, if not all, of the genes encoding heme uptake systems that mediate the acquisition of free environmental heme and/or hemoproteins^20^ (Figure S1). Heme uptake systems comprise at least a membrane‐bound receptor (TonB‐dependent receptor [TBDT] in Gram‐negative bacteria) and an ABC transporter^21^, ^22^. This gene organization feature suggests that the primary function of bacterial HOs is to degrade exogenous heme as an iron source, either free or bound to polypeptide, especially in pathogens that encounter large amounts of hemes/hemoproteins of their hosts^23^, ^24^. We speculated that these HOs associated with heme uptake systems may play the role of degrading heme *c*, because all cyts *c* are extracytoplasmic proteins.

A global phylogenomic survey revealed a broad taxonomic distribution of HO lineages spanning the tree of life (Figure 1A). Although ChuW and HemS are largely restricted to the Gram‐negative phylum *Pseudomonadota*, all of these HO types can be found in both *Bacillota* and *Pseudomonadota*, suggesting that the evolution of HOs has been rather complex, presumably due to the presence of multiple ancestors, high susceptibility to mutations, and extensive horizontal transfer^25^, ^26^. The extensive phylogenetic overlap also implies that the ability to degrade both heme *b* and heme *c* might be dictated by specific structural adaptations rather than certain taxonomic lineages. Guided by this insight, we shifted our focus to the protein folds and active site architectures.

**Figure 1:**
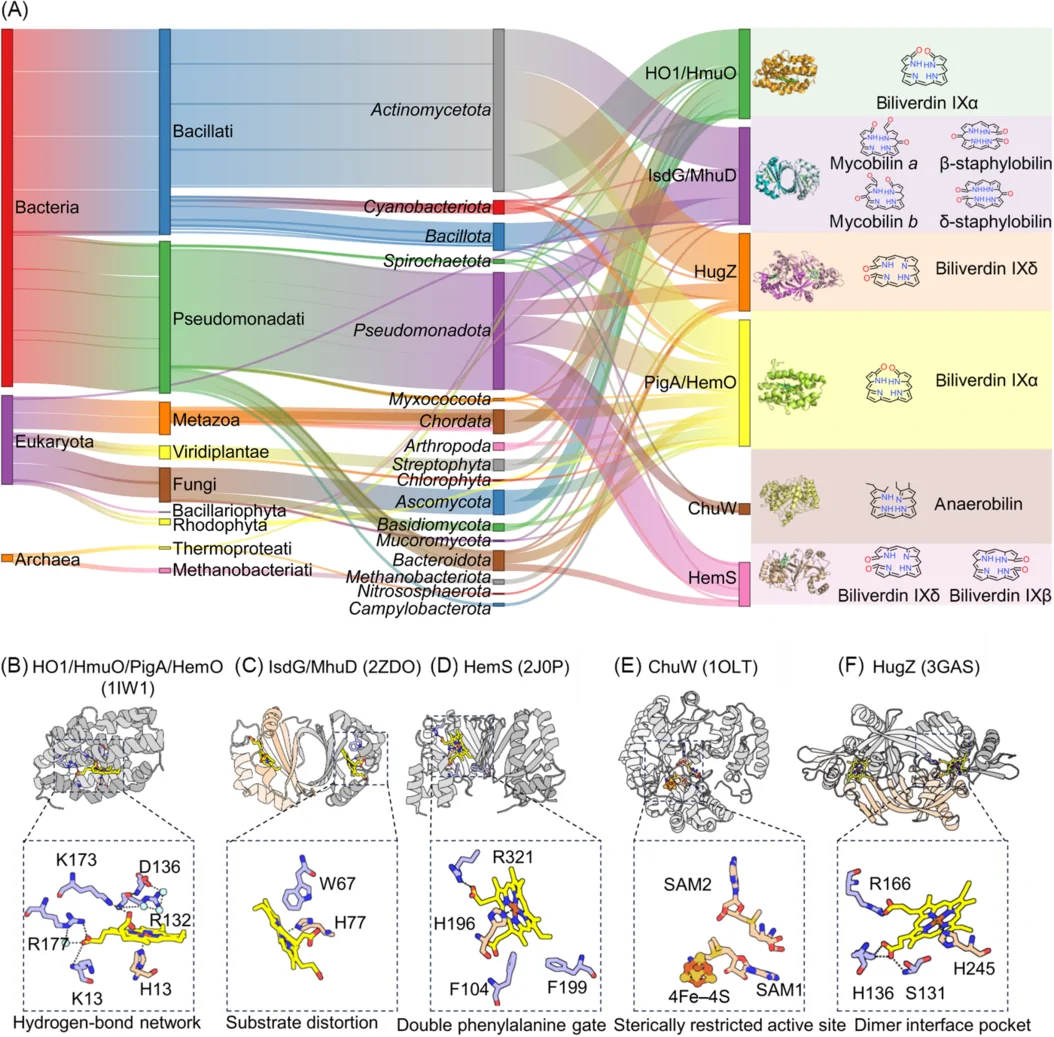
Taxonomic distribution, heme‐cleavage products, and structural diversity of the heme oxygenase (HO) superfamily. (A) Integrated overview of the taxonomic distribution, representative structures, and heme‐cleavage products of six types of HOs. A Sankey diagram maps the prevalence of HO genes across biological domains and phyla to specific families defined by InterPro signatures: HO1/HmuO (IPR002051), IsdG/MhuD (IPR050404), HugZ (IPR052019), PigA/HemO (IPR016053), ChuW (IPR026332), and HemS (IPR007845). Bandwidth is proportional to sequence abundance. Representative crystal structures and corresponding heme cleavage products are annotated to highlight the correlations between protein fold and catalytic regiospecificity. (B–F) Structural diversity of heme‐binding pockets and catalytic centers in representative HOs. The upper images display the overall 3D architectures, while the lower insets highlight the distinct local coordination environments and key interacting residues. The HO1/HmuO/PigA/HemO type (B), represented by HmuO (PDB: 1IW1), features a complex hydrogen‐bond network stabilizing the heme cofactor. The IsdG/MhuD type, represented by IsdG (PDB: 2ZDO) (C), induces severe substrate distortion of the porphyrin ring to facilitate cleavage. The HemS type (PDB: 2J0P) (D) utilizes a “double phenylalanine gate” to sequester the heme. The ChuW type (E) features a sterically restricted active site. Due to the lack of an available experimental structure for ChuW, the highly homologous HemN (PDB: 1OLT) is utilized as a representative structural surrogate, highlighting the conserved spatial arrangement of the [4Fe‐4S] cluster and two S*‐*adenosyl‐l‐methionine (SAM) molecules. The HugZ type (PDB: 3GAS) binds the heme cofactor within a distinct dimer interface pocket (F).

Extensive structural studies have established that the specific architectures correlate tightly with distinct heme cleavage products (Figure 1A). Canonical α‐helical HOs, including mammalian HO1 and its bacterial homologs such as HmuO, PigA, and HemO, share a conserved all‐α fold, in which the heme substrate is deeply buried within a compact active‐site cavity^27^, ^28^, ^29^. Long proximal and distal α‐helices tightly enclose the porphyrin macrocycle, with a conserved proximal histidine serving as the axial iron ligand and an ordered distal hydrogen‐bond network enforcing strict meso‐carbon regioselectivity toward free heme *b* (Figure 1B)^30^, ^31^. As a result, the architectures of the canonical α‐helical HOs impose severe steric constraints and confer strong substrate specificity, suggesting that they are unlikely to degrade heme *c*. IsdG/MhuD‐type HOs adopt an antibiotic biosynthesis monooxygenase (ABM) fold^32^, ^33^ and use a distinct catalytic strategy centered on substrate distortion^34^, ^35^. This mechanism, which critically depends on the ability of the heme macrocycle to undergo substantial out‐of‐plane deformation, is incompatible with the increased macrocycle rigidity resulting from the covalent attachment of heme *c* to the polypeptide via dual thioether bonds (Figure 1C)^36^, ^37^. Although both HemS‐ and ChuW‐type HOs catalyze heme degradation under anaerobic conditions^38^, ^39^, structural constraints preclude them from accommodating heme *c*. In HemS, this restriction arises from a double phenylalanine gate positioned between the heme and NADH binding sites (Figure 1D)^40^. Similarly, ChuW‐type radical S‐adenosyl‐l‐methionine (SAM) enzymes share a highly conserved structural topology with HemN (coproporphyrinogen III oxidase), whose crystallographic structure indicates that the substrate‐binding pocket is sterically restricted by a bulky 4Fe–4S cluster and two SAM cofactors (Figure 1E), intrinsically confining the cavity to enclose specific substrates and shield the reaction intermediate^41^, ^42^.

The final group of HOs belongs to the HugZ family, which are characterized by a unique split β‐barrel architecture with a heme‐binding pocket at the dimer interface that allows the production of δ‐biliverdin strictly (Figure 1F)^25^, ^43^. This structural feature creates an unusually open, modular active site that relaxes geometric constraints for broad substrate accommodation, seemingly conferring upon the HugZ family HOs the potential to degrade both heme *b* and heme *c*.

### Phylogenetic analysis of the HugZ superfamily reveals the Core‐only type with dual‐substrate potential

To investigate the intrinsic plasticity of the HugZ split β‐barrel architecture for diverse heme substrates, we constructed a sequence similarity network (SSN) by integrating all known HO sequences (∼16,400) from two superfamilies: the HugZ‐like (IPR037119) and the HutZ‐like (IPR014419) using EFI‐EST^44^, ^45^. The SSN, comprising sequences predominantly from bacteria and plants with a small number of archaeal representatives, illustrates a complex topological landscape partitioned into two major clusters (Figure 2). Cluster I predominantly includes the HugZ (IPR026324) and HugZ‐like (IPR037119) families, whereas Cluster II is composed of the HutZ‐like proteins exclusively. Within Cluster I, the primary sub‐cluster encompasses HOs from highly diverse taxonomic lineages, indicative of extensive evolutionary expansion. In addition, Cluster I is further fractionated into multiple cohesive sub‐clusters, such as the canonical HugZ family (IPR026324). The absence of interconnections between these sub‐clusters underscores substantial sequence divergence. Surprisingly, *c*HOA from *Paracoccus denitrificans* (*Pdc*HOA), traditionally annotated as a HutZ‐like HO, is embedded within Cluster I, implying the complex evolutionary boundaries between these families. Cluster II shows a similar topology, being dominated by a single major core surrounded by isolated, minor sub‐clusters.

**Figure 2:**
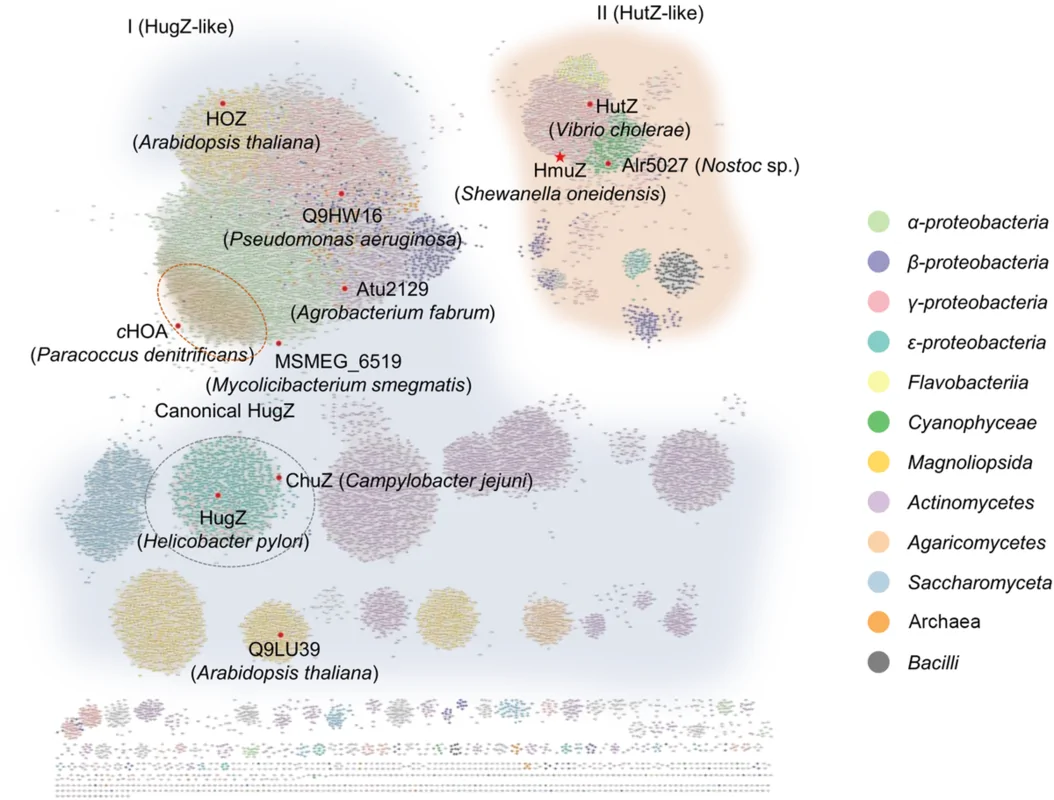
Sequence similarity network (SSN) of the HugZ superfamily. The SSN was constructed using the (Enzyme Similarity Tool) EFI‐EST from 16,400 sequences belonging to the HugZ‐like (IPR037119) and HutZ‐like (IPR014419) families. Nodes are colored according to taxonomy, and red dots denote proteins with solved crystal structures (PDB). The red star marks HmuZ from *Shewanella oneidensis*, the principal protein investigated in this study. The network can be broadly divided into two main clusters. Cluster I (HugZ‐like), highlighted with a blue background, contains the canonical HugZ family, which is outlined in gray. Cluster II (HutZ‐like), highlighted with a orange background, contains a subgroup outlined in red.

To further resolve the evolutionary landscape of the HugZ superfamily, a comprehensive phylogenetic analysis of Clusters I and II was carried out, revealing three distinct architectural types: the Core‐only, the N‐fusion, and the C‐extension (Figures 3 and S2). The Core‐only type represents the most compact form at approximately 180 amino acids (aa), as typified by HutZ from *Vibrio cholerae* (*Vc*HutZ)^46^, ^47^, whereas the N‐fusion and C‐extension types generally exceed 250 aa in length due to the integration of a fused additional domain, exemplified by HugZ from *Helicobacter pylori* (*Hp*HugZ) and HOZ from *Arabidopsis thaliana*, respectively^43^. Evolutionary models propose that the C‐extension type represents the ancestral state, yielding the Core‐only type through domain loss and the N‐fusion type via subsequent refusion events^25^. Eukaryotic HugZ‐type HOs (predominantly from *Magnoliopsida*), mostly belonging to the C‐extension type, are further characterized by a total length exceeding 300 aa due to extended, non‐conserved N‐terminal loops, which likely function as organelle‐targeting transit peptides^48^. Bacterial HOs within the HugZ superfamily encompass all three structural architectures, displaying a highly intermingled phylogenetic distribution. C‐extension‐type HOs are primarily present in *γ‐* and *β‐proteobacteria*, as well as *Actinomycetes*, whereas the N‐fusion‐type HOs are largely restricted to *ε‐proteobacteria*. In contrast, the Core‐only‐type HOs can be found in a large number of bacterial phyla, indicating that HOs of this type have been evolutionarily honed for heme degradation in diverse genetic backgrounds. Given that the wide distribution of the Core‐only‐type HOs perfectly matches the abundance of cyts *c* in bacteria, we hypothesized that HOs capable of degrading both heme *b* and heme *c* most likely belong to the Core‐only type.

**Figure 3:**
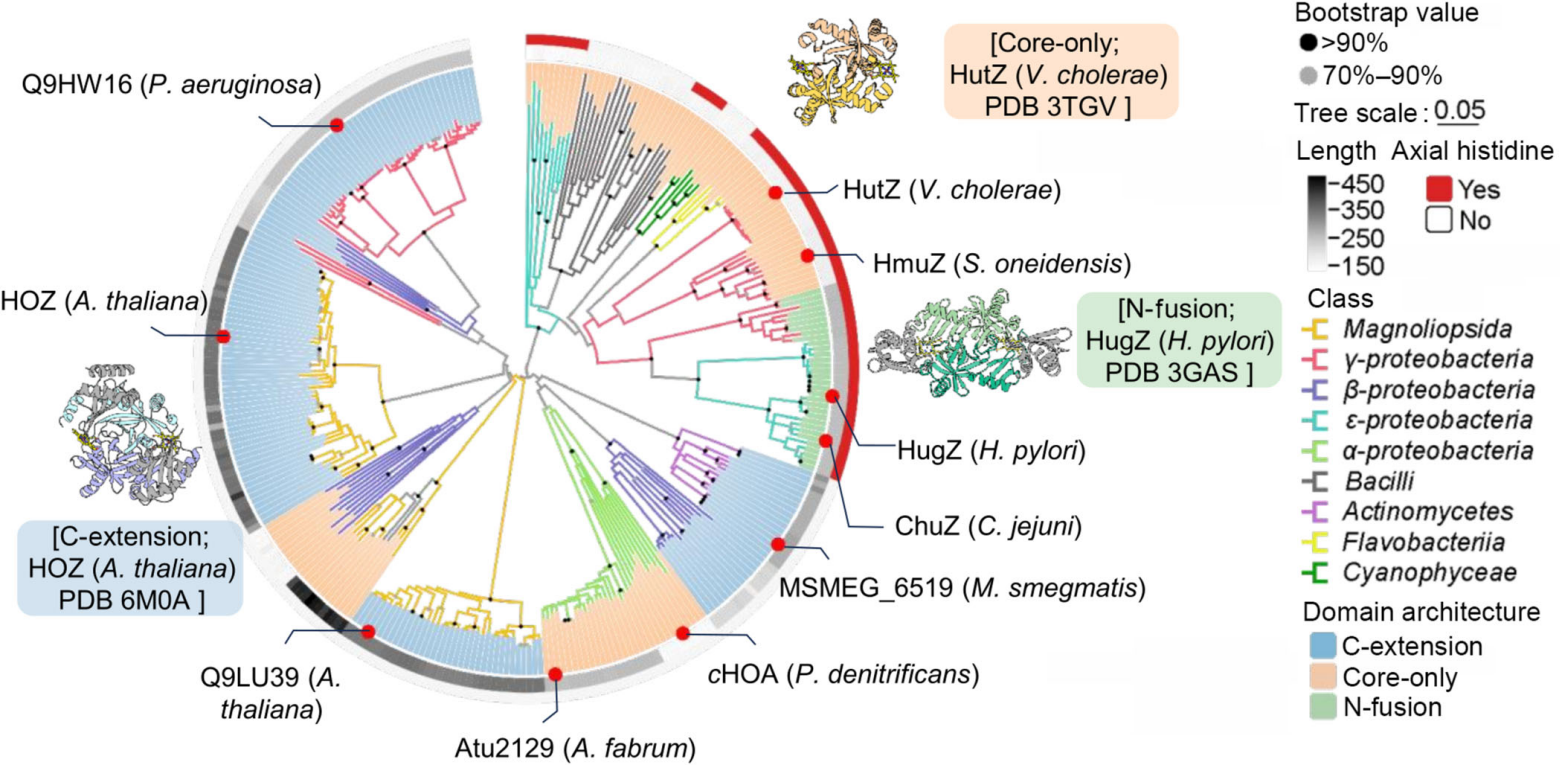
Phylogenetic analysis of the HugZ‐type HO superfamily based on core domain sequences. The neighbor‐joining (NJ) tree was constructed using 339 representative sequences from SSN in Figure 2 aligned by Multiple Alignment using Fast Fourier Transform (MAFFT). Annotations map four layers of taxonomic and structural information to the tree topology according to the color keys. Taxonomic lineages are color‐coded, highlighting major groups. Structural organization is categorized into three distinct domain architectures, namely, C‐extension (blue), Core‐only (orange), and N‐fusion (green). Protein sequence length is visualized using a grayscale gradient that spans from white for shorter sequences (~150 aa) to black for longer sequences (~450 aa). The presence of the conserved distal axial histidine ligand is highlighted in red. Bootstrap values from 1000 replicates are indicated by colored circles at the nodes (black, >90%; gray, 70%–90%).

### HmuZ of *S. oneidensis* is an HO active against heme *b* and heme *c*

To test the hypothesis that Core‐only type HO can degrade both heme *b* and heme *c*, HmuZ of the Core‐only type from *S. oneidensis* (*So*HmuZ), which is a *γ‐proteobacterium* particularly rich in cyts *c*, was selected. This enzyme is proposed to be the only HO responsible for degrading endogenous heme *b* into biliverdin, conferring HmuZ‐overexpressing cultures with a distinct green coloration (Figure S3A)^49^, ^50^. In *S. oneidensis*, the genes for heme uptake are organized into three operons: *hmuVUT* encoding an ABC transporter, *exbDB‐tonB* comprising the energy‐transducing system, and *hmuAXZ* encoding TBDT, heme‐binding, and degrading proteins, respectively (Figure S3B). By modulating heme *b* levels, the absence and overproduction of HmuZ resulted in increased and decreased overall heme *c* content, respectively (Figure S3C), as reported before^49^, ^51^.

For biochemical substantiation, a C‐terminal His_6_‐tagged recombinant *So*HmuZ was expressed and purified from *Escherichia coli*. Purified *So*HmuZ migrated at approximately 22.5 kDa on SDS‐PAGE, which was further confirmed by Western blotting (Figures 4A and S3D). UV–visible spectroscopy of the hemin–*So*HmuZ complex revealed a Soret peak at 420 nm and α/β‐bands at 543/577 nm, characteristic of heme‐binding proteins (Figure 4A). In hemin titration assays, incremental addition of heme *b* (hemin) to the apo‐*So*HmuZ caused a progressive increase in Soret band absorbance, reaching saturation at a 1:1 molar stoichiometry (Figure 4B). As the structurally characterized HOs of the HugZ superfamily exist as dimers^43^, ^47^, ^52^, this stoichiometry indicates that the *So*HmuZ dimer binds two heme molecules. Fluorescence quenching confirmed the 1:1 binding ratio yielding dissociation constants (*K*_d_) of 8.4 ± 1.2 μM at 37°C and 9.1 ± 2.2 μM at 30°C (Figure S4A–D and Table S1). Similarly, spectroscopic characterization and differential absorption titration demonstrated that *So*HmuZ binds microperoxidase‐11 (MP‐11, an 11‐amino‐acid fragment containing covalently bound heme *c*) with a strict 1:1 stoichiometry, accompanied by a detectable Soret band shift to 408 nm (Figure S5A,B).

**Figure 4:**
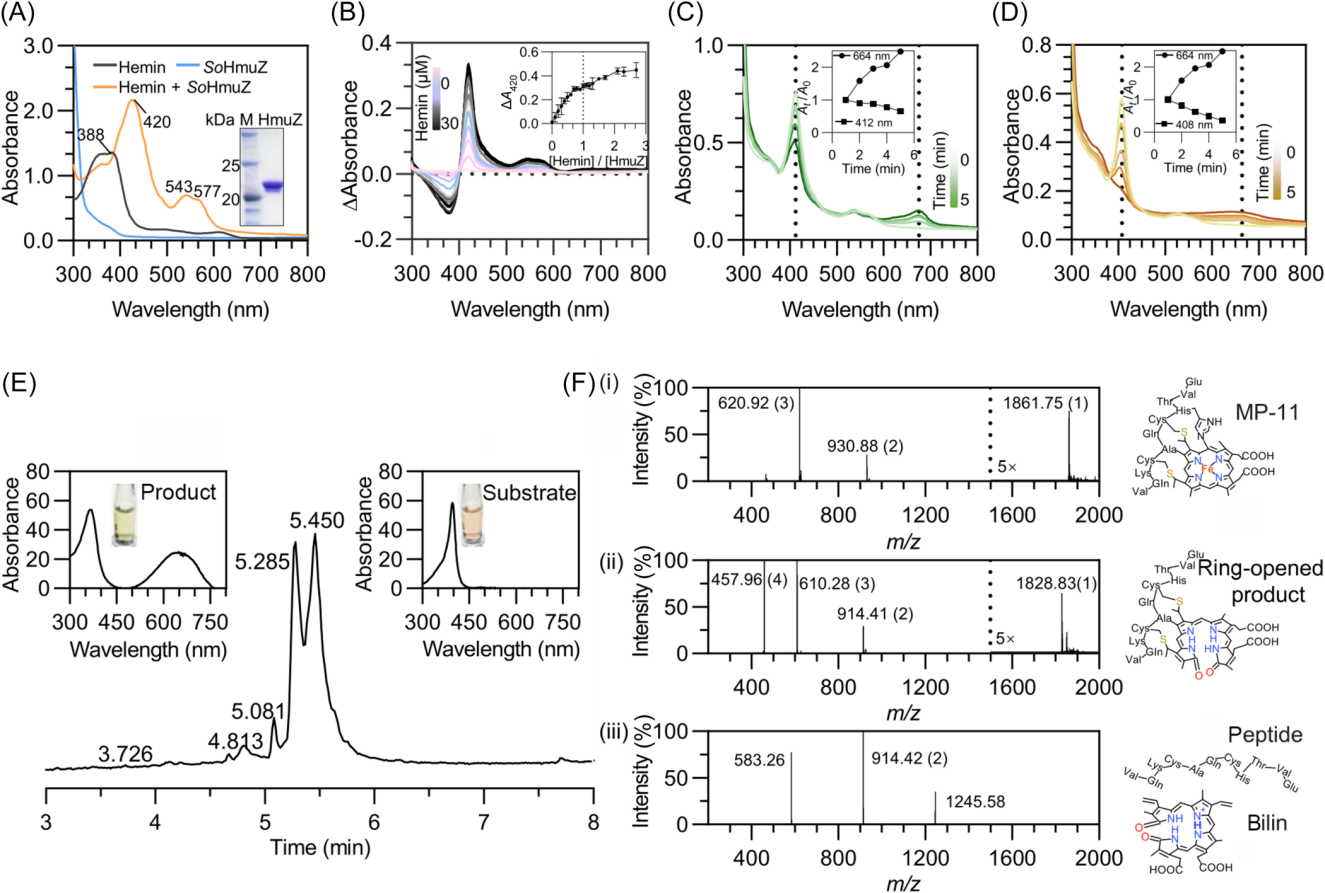
*So*HmuZ is a dual‐substrate HO active against heme *b* and heme *c*. (A) UV–visible absorption spectra of hemin, *So*HmuZ, and the hemin–*So*HmuZ complex. Inset: analysis of purified *So*HmuZ protein by Coomassie blue‐stained SDS‐PAGE. (B) Hemin‐binding activity of *So*HmuZ monitored by differential absorption spectroscopy. Spectral changes are shown during the titration of *So*HmuZ (10 μM) with hemin in 20 mM Tris‐HCl, 150 mM NaCl (pH 7.0) supplemented with 2 μM catalase, and 20 μM BSA. The characteristic positive absorption peaks indicate hemin binding. Inset: the corresponding titration curve monitored at 420 nm. Error bars represent standard deviations (SD) from three independent replicates (*n* = 3). (C, D) Time‐resolved UV–visible absorption spectra of *So*HmuZ‐catalyzed degradation of 20 μM hemin (C) and 10 μM MP‐11 (D) in the presence of 10 mM ascorbate, 2 μM catalase, and 20 μM BSA in 20 mM Tris‐HCl and 150 mM NaCl (pH 7.0). Insets show the time course of the Soret band intensity changes (substrate complex, squares; product, circles), with the color gradient indicating reaction progress over 5 min. (E) High‐Performance Liquid Chromatography (HPLC) chromatogram of the *So*HmuZ reaction mixture. Insets show the UV–visible absorption spectra and photographic images of the fractions eluted at 5.285 min (product) and 5.450 min (substrate). (F) Liquid chromatography‐tandem mass spectrometry (LC–MS/MS) characterization of the MP‐11 degradation product. Representative electrospray ionization (ESI) mass spectra of the indicated chromatographic peaks are shown. (i) Full MS scan of the intact MP‐11 substrate (retention time: 5.450 min); (ii) the *So*HmuZ‐generated product (retention time: 5.285 min), with high‐mass regions magnified 5×; and (iii) MS/MS fragmentation spectrum of the product precursor ion. Charge states are provided in parentheses. This high‐resolution mass spectrometric analysis confirms the formation of a peptide‐linked biliverdin.

We subsequently carried out heme degradation assays using heme *b* (hemin), commercially available intact equine heart cyt *c*, and its proteolytic product MP‐11. Upon incubating *So*HmuZ with ascorbate and hemin, the Soret band at 412 nm decreased over time, concomitant with the emergence of characteristic peaks between 650 and 700 nm (Figure 4C), which primarily reflect transient accumulation of the verdoheme^53^. Under optimized conditions, the reaction achieved a greater than 30% reduction of the Soret band at 412 nm within 5 min (Figure 4C). The catalytic reaction of *So*HmuZ with MP‐11 resulted in a 60% reduction of the Soret band at 408 nm within 5 min under identical conditions (Figure 4D), suggesting that *So*HmuZ is similarly effective against heme *b* and heme *c*. Degradation of either hemin or MP‐11 was not detected in control assays lacking either the *So*HmuZ enzyme or the electron donor ascorbate (Figure S6A–D), confirming that the enzyme catalyzes the reactions in an ascorbate‐dependent manner. Liquid chromatography‐tandem mass spectrometry (LC‐MS/MS) analysis revealed the product of the *So*HmuZ reaction with MP‐11 to be a biliverdin molecule conjugated to the peptide (Figure 4E,F). In contrast, under identical conditions, no spectral changes for the intact equine heart cyt *c* were observed, implying that purified *So*HmuZ could not degrade heme *c* from unprocessed cyt *c* (Figures 5A and S6E,F).

**Figure 5:**
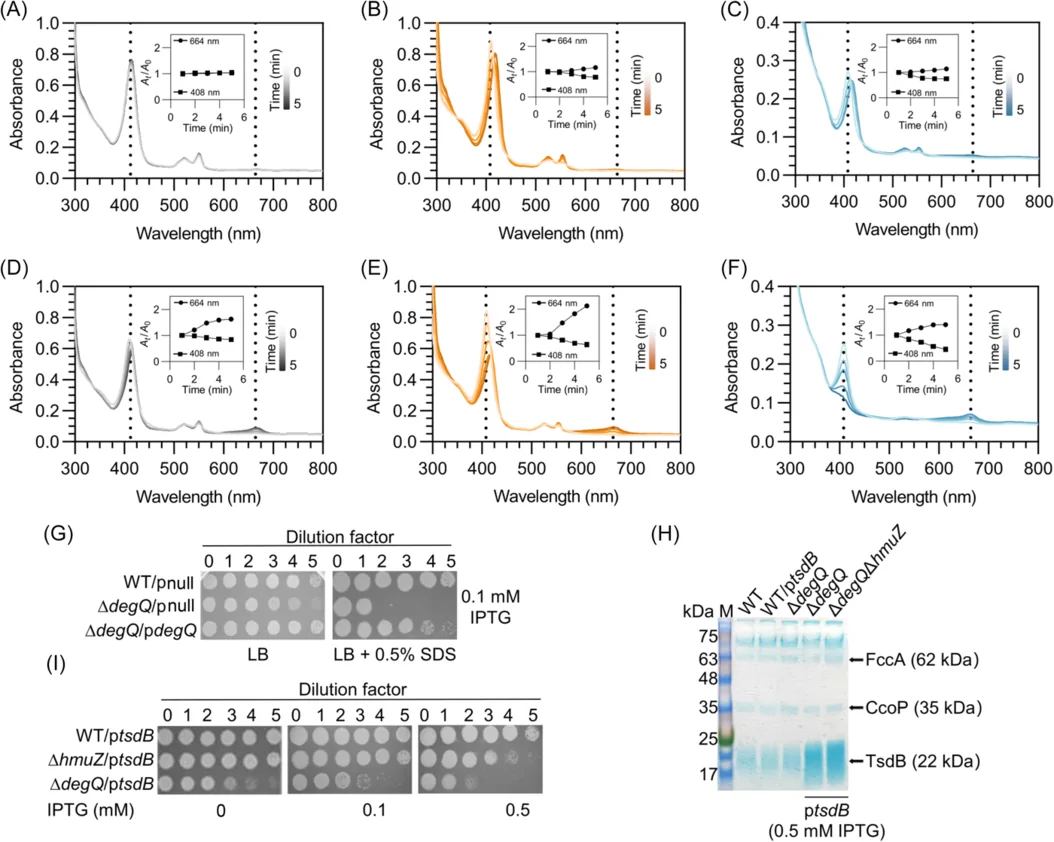
Proteolytic preprocessing is required for *So*HmuZ‐mediated cytochrome *c* degradation. (A–F) Time‐resolved UV–visible absorption spectra of *So*HmuZ reacting with equine heart cytochrome *c* (gray), ScyA (orange), or TsdB (blue). The substrates in (A–C) are untreated and those in (D–F) are trypsin‐pretreated. Insets show Soret band changes over time (substrate, squares; product, circles), indicating accelerated turnover upon proteolytic preprocessing. (G) Deletion of *degQ* increases SDS sensitivity. Droplet assays of WT/pnull, Δ*degQ*/pnull, and Δ*degQ*/p*degQ* on LB agar ± 0.5% SDS. (H) Heme‐staining analysis of total proteins from the wild type (WT) and indicated mutant strains expressing TsdB. Cells were induced with IPTG as indicated. Arrows denote endogenous *c*‐type cytochromes FccA and CcoP, and overexpressed TsdB. (I) TsdB overexpression preferentially impairs growth in the Δ*degQ* background. Droplet assays of WT, Δ*hmuZ*, and Δ*degQ* strains carrying p*tsdB* under increasing IPTG induction concentration are shown.

### DegQ is a prerequisite for SoHmuZ‐mediated heme *c* degradation

To substantiate that proteolytic cleavage is a prerequisite for heme *c* degradation by *So*HmuZ, we tested two native cyt *c* proteins: monoheme cyt *c*_5_ (ScyA)^50^ and diheme cyt *c*_4_ (TsdB)^54^. Consistent with the results for equine heart cyt *c*, neither ScyA nor TsdB was appreciably degraded by HmuZ (Figure 5B,C). In contrast, effective degradation of both forms of cyt *c* was observed after trypsin treatment (Figures 5D–F and S7A–F).

Proteolysis of cyt *c* proteins most likely occurs in the periplasm. In *S. oneidensis*, the major protease responsible for periplasmic protein quality control is DegQ^55^. We thus constructed a *degQ* knockout (Δ*degQ*) strain, which shows slightly slower growth on LB medium and hypersensitivity to SDS (Figure 5G), phenotypes attributed to aberrant quality control of outer‐membrane proteins^56^. Examination of cyt *c* abundance by heme staining revealed significant cyt *c* accumulation in the Δ*degQ* strain relative to the WT, despite equivalent total protein levels (Figures 5H and S8). This accumulation was particularly evident in cells overproducing TsdB within the Δ*degQ* compared to the WT, implying that DegQ preferentially degrades aberrantly expressed cyts *c* (Figure 5H). Furthermore, excess TsdB severely impaired the growth of Δ*degQ*, whereas the inhibitory effect was negligible in the WT and less pronounced in *ΔhmuZ* (Figure 5I). Collectively, these data indicate that (i) cyt *c* proteins are processed in the periplasm predominantly by DegQ, which is the prerequisite for *So*HmuZ‐mediated degradation and (ii) the recycling of surplus cyt *c* is crucial for cellular fitness.

### A balanced heme‐binding pocket enables the bifunctional competence of *So*HmuZ

To elucidate the structural basis for heme *b* and heme *c* degradation by *So*HmuZ, we compared its structure with representatives of the other two architectural types. The homodimeric structure of *So*HmuZ in complex with heme *b* was predicted using AlphaFold 3^57^, yielding a high‐confidence model that closely aligns with the experimentally solved *Vc*HutZ (PDB: 3TGV; RMSD = 0.712 Å) and *Hp*HugZ (PDB: 3GAS; RMSD = 1.281 Å) (Figure S9A–D). This overall structural conservation provides a framework to delineate functional divergence across the superfamily.

Given the divergent heme‐binding modes and regulatory roles of the C‐extension‐type HOs (Supplementary text in the Supporting Information), we focused our comparative analysis on the Core‐only‐ and N‐fusion‐type HOs. Superposition of the representative structures reveals nearly identical spatial configurations, featuring a conserved dimeric β‐barrel core and an interfacial heme‐binding pocket (Figure 6A). In the representative *So*HmuZ model, the heme is coordinated by a highly conserved pocket comprising S60, H65, R94, F135, F151, and H172 (Figure 6B). Specifically, R94 interacts with a heme‐bound water molecule rather than the heme directly. As demonstrated in *Vc*HutZ and *Hp*HugZ, this configuration is critical for proton transfer to the Fe(III)–OO^−^ intermediate during ascorbate‐dependent degradation^43^, ^58^. Concurrently, H65 and S60 stabilize the heme propionate groups via hydrogen bonding, potentially modulating subunit interactions within the R94–H172 network^47^, ^59^. Furthermore, F135 and F151 secure the porphyrin ring through π–π stacking and van der Waals interactions, respectively, ensuring optimal hydrophobic stability and precise heme positioning^43^, ^47^. Collectively, this coordinated network maintains the structural and functional integrity of the active site, highlighting a conserved heme‐binding architecture across the Core‐only‐ and N‐fusion‐type HOs.

**Figure 6:**
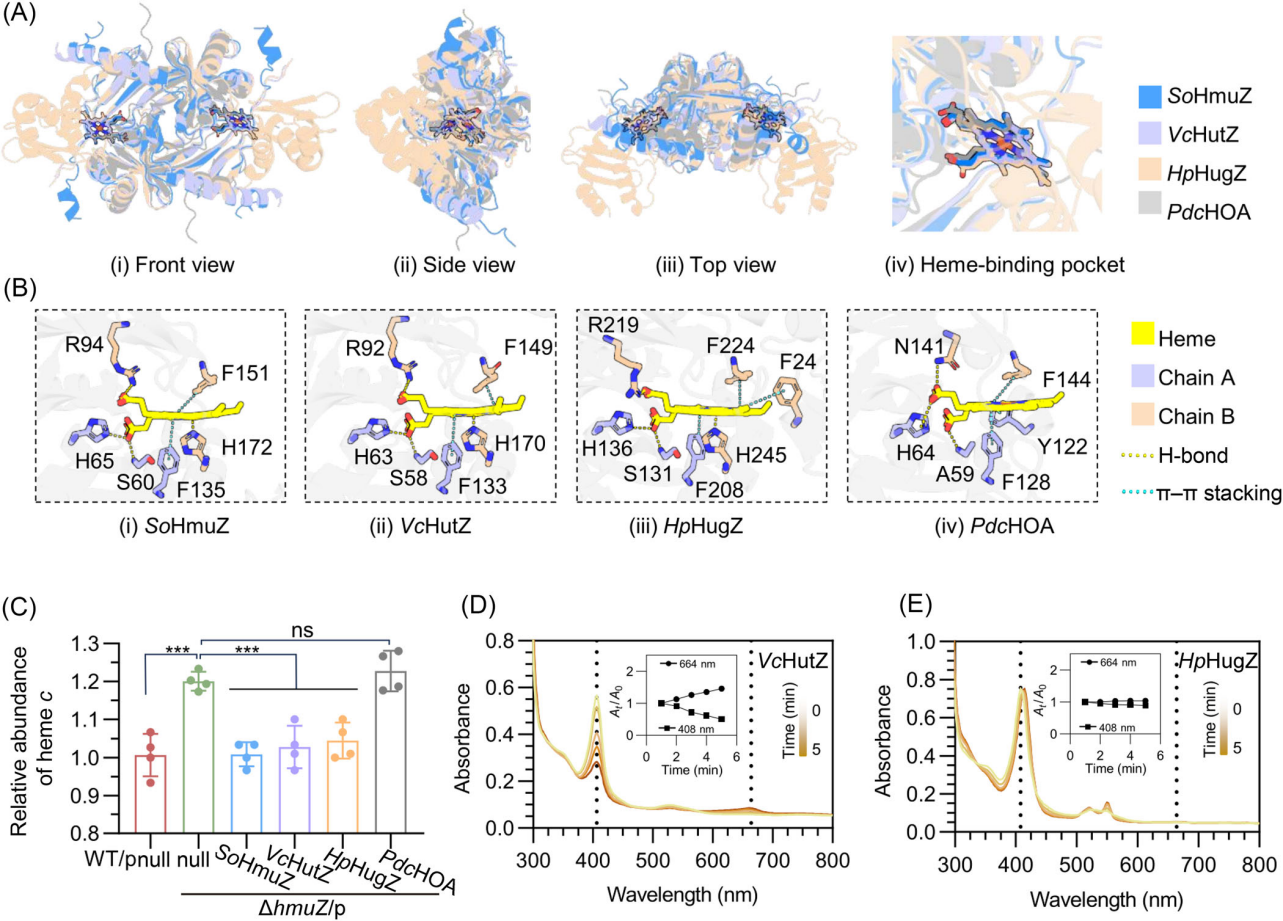
Structural comparison and functional analysis of *So*HmuZ homologs. (A) Structural superposition of *So*HmuZ with its homologs (*Hp*HugZ, *Vc*HutZ, and *Pdc*HOA), highlighting the conserved β‐barrel core and heme‐binding pockets. (B) Close‐up view of key residue interactions within the heme‐binding pockets. (C) Effect of homologous protein complementation on heme *c* levels in *S. oneidensis*. The relative abundance was normalized to the total protein concentration and calculated relative to the WT/pnull. Data are presented as mean ± SD (*n* = 4). Statistical significance was determined by one‐way ANOVA, with the Δ*hmuZ*/pnull mutant serving as the reference group for comparisons. ***p < 0.001; ns, not significant. (D, E) Time‐resolved UV–visible spectra of MP‐11 (10 μM) degradation catalyzed by *Vc*HutZ (D) and *Hp*HugZ (E) in the presence of 10 mM ascorbate, 2 μM catalase, and 20 μM BSA in 20 mM Tris‐HCl and 150 mM NaCl (pH 7.0). Insets show the time course of Soret band intensity changes (substrate, squares; product, circles), with the color gradient indicating the reaction progress over 5 min.

To test the degradation specificity of these representative HOs, we expressed each of them in *S. oneidensis*. Similar to *So*HmuZ, the overproduction of *Vc*HutZ and *Hp*HugZ, but not *Pdc*HOA, significantly lowered overall heme *c* levels (Figure 6C), suggesting that *Vc*HutZ and *Hp*HugZ may degrade heme *b*, thereby depleting the precursor pool required for heme *c* biosynthesis. However, *in vitro* assays with purified proteins confirmed that *Vc*HutZ, but not *Hp*HugZ, degrades heme *c* (Figure 6D,E). We then proposed that *Hp*HugZ may not be able to degrade heme *c* because its N‐fusion domain likely acts as a steric barrier preventing access to the bulkier heme *c*. This proposal was supported by molecular simulations in which forced docking of MP‐11 induces an opening motion in the N‐fusion domain, indicating a substantial steric clash with the bulkier substrate (Figure S9E).

### Structural precision of the distal catalytic network dictates proton transfer efficiency

To provide in‐depth insights into the functional plasticity of *So*HmuZ, key residues were subjected to site‐directed mutagenesis, and kinetic profiling of the resulting *So*HmuZ variants against both hemin and MP‐11 was performed. For hemin degradation, the R94A mutation significantly reduced the maximal reaction velocity (*V*_max_) and the corresponding turnover efficiency (*k*_cat_) to 42% of the WT level without a concomitant loss of substrate affinity (the apparent half‐saturation constant, *K*_0.5_, remained comparable at 114%) (Table 1 and Figure 7A). To elucidate the structural basis for the reduction in *V*_max_ and *k*_cat_, MD simulations of the hemin‐bound complex were performed. The trajectories revealed that the R94A substitution disrupted the local distal environment, with the distance between residue 94 and the heme group increasing from ∼0.3 nm in WT to ∼0.8 nm in the mutant, thereby disrupting the native interaction network (Figure 7B). Concurrently, the Hill coefficient (*n*) decreased, supporting reduced cooperativity (2.15 vs. 2.87) (Table 1). These data corroborate R94 as a critical participant in the rate‐limiting step, presumably the proton transfer required for degradation, while the decrease in *n* highlights its role in regulating allosteric subunit coordination^53^. In addition, the R94A mutation also caused increased fluctuations in water occupancy within the active site (Figure 7C), without significantly affecting interface stability (Figure 7D).

**Table 1:** Catalytic activity of the *So*HmuZ variants.

Substrate	Variant	*V*_max_ (μM/min)	*K*_0.5_ (μM)	*n* (Hill coefficient)	*k*_cat_ (min^−1^)
Hemin	WT	0.193 ± 0.027	4.31 ± 0.28	2.87 ± 0.72	0.386 ± 0.054
	T29V	0.108 ± 0.011	4.19 ± 0.33	1.97 ± 0.41	0.216 ± 0.022
	R94A	0.081 ± 0.009	4.92 ± 0.38	2.15 ± 0.52	0.162 ± 0.018
	H172A	0.004 ± 0.001	N/A	N/A	0.008 ± 0.000
	L132A + D134L + H172A	0.062 ± 0.007	4.25 ± 0.31	1.89 ± 0.38	0.124 ± 0.014
MP‐11	WT	0.016 ± 0.001	3.22 ± 0.51	2.05 ± 0.32	0.032 ± 0.002
	T29V	0.022 ± 0.002	1.55 ± 0.32	1.52 ± 0.21	0.044 ± 0.004
	R94A	0.014 ± 0.001	1.81 ± 0.41	1.43 ± 0.24	0.028 ± 0.002
	H172A	0.010 ± 0.001	2.53 ± 0.64	1.21 ± 0.31	0.020 ± 0.002
	L132A + D134L + H172A	0.032 ± 0.003	1.21 ± 0.34	1.38 ± 0.22	0.064 ± 0.006

*V*_max_, *K*_0.5_, *n*, and *k*_cat_ values for the degradation of hemin (heme *b*) and microperoxidase‐11 (MP‐11, representing heme *c*) by the *So*HmuZ and its site‐directed variants. Values represent the mean ± standard deviation (SD) of three independent replicates (*n* = 3). N/A indicates that the parameters could not be reliably fitted to the Hill equation due to extremely low catalytic activity. *K*_0.5_, apparent half‐saturation constant; *k*_cat_, turnover number; *n*, Hill coefficient; *V*_max_, maximum reaction velocity.

**Figure 7:**
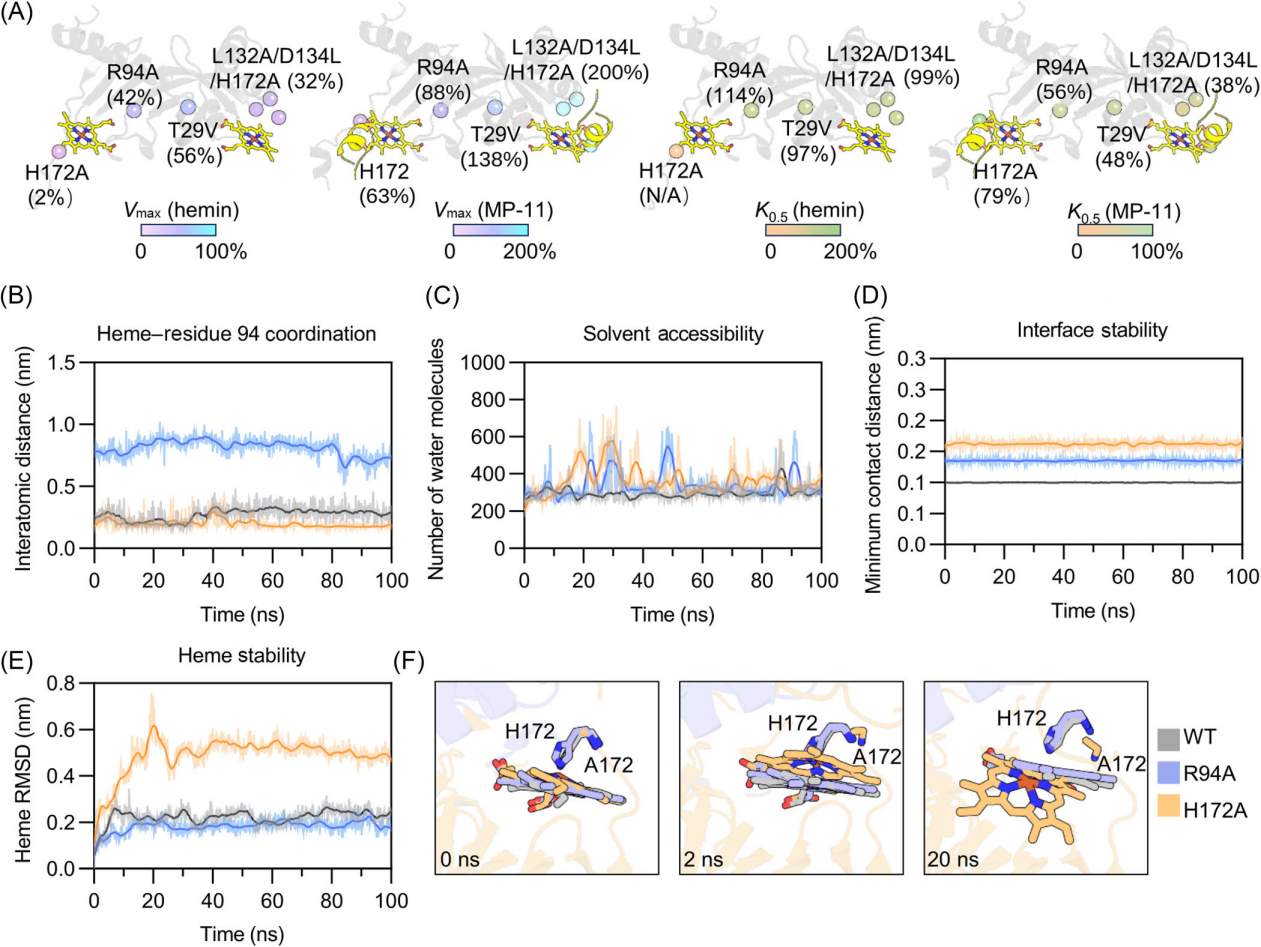
Structural mapping of mutational effects and conformational dynamics of the *So*HmuZ substrate pocket. (A) 3D structural mapping of the relative kinetic parameters (*V*_max_) and the apparent half‐saturation constant (*K*_0.5_) for hemin and MP‐11. The mutated residues are represented as spheres and colored according to their relative percentages compared to the WT. N/A indicates no detectable activity. (B–E) 100 ns molecular dynamics (MD) simulation trajectories for the WT (gray), R94A (blue), and H172A (orange). The plots show the time evolution of the interatomic distance representing heme–residue 94 coordination (B); solvent accessibility(C), measured by the number of water molecules within a 5 Å radius of the heme; interface stability (D), indicated by the minimum contact distance; and heme stability (E), measured by the root‐mean‐square deviation (RMSD) of the heme group. (F) Representative structural snapshots at 0, 2, and 20 ns, visually illustrating the severe displacement of heme in the H172A mutant (orange) relative to the WT (gray) and R94A (blue).

However, the R94A variant displayed distinct behavior toward MP‐11 (Table 1 and Figure 7A). While the reduction in both *V*_max_ and *k*_cat_ was found to be much less substantial (to 88%), cooperativity was notably attenuated (*n*: 1.43 vs. 2.05) and binding affinity was paradoxically enhanced (*K*_0.5_ decreased to 56%). We postulated that the hydrophilic peptide moiety may provide an alternative local environment or proton source, which is in line with the enhanced solvent accessibility observed in our simulations (Figure 7C), thereby diminishing the enzyme's reliance on the R94‐mediated proton transfer network (Figure 7B). To further elucidate this network, we mutated T29, a residue structurally adjacent to R94 and acting as a proton source by stabilizing a catalytic water cluster near the Fe(III)–OO^−^ intermediate^58^, ^60^, ^61^. Consistently, the *V*_max_ of the T29V mutant on heme *b* decreased significantly to 56%, accompanied by a noticeable decrease in *n* (1.97 vs. 2.87). Strikingly, toward MP‐11, the T29V mutant showed functional potentiation, with both *V*_max_ and *k*_cat_ increasing to 138%, while *n* decreased (1.52 vs. 2.05) (Table 1 and Figure 7A). This difference likely arises because the WT T29 hydroxyl forms non‐productive hydrogen bonds with the MP‐11 peptide tail or introduces steric hindrance, and its removal by T29V effectively relieves these clashes.

### Proximal anchoring governs structural stability and substrate selectivity

A remaining puzzle is why *Pdc*HOA is unable to degrade heme *b* ^20^. Phylogenetic mapping of the proximal histidine reveals a lineage‐specific distribution rather than strict domain‐based conservation. Phylogenetically, sequences containing this axial histidine cluster densely within a specific major evolutionary clade (Figures 3 and S2). Specifically, within the Core‐only‐type HOs, this residue is predominantly distributed across selected genera, including *Vibrio*, *Shewanella*, *Paenibacillus*, *Sulfurospirillum*, and *Chryseobacterium*. In these lineages, this histidine residue acts as a rigid catalytic anchor, enabling the efficient degradation of heme *b*. Furthermore, this site is highly conserved in N‐fusion‐type HOs, primarily within *Campylobacter*, *Helicobacter*, and *Rodentibacter*. In these enzymes, steric shielding by the N‐terminal domain creates a spatially restricted catalytic pocket, preventing the entry of the bulkier heme *c* (Figure S9E). In contrast, this typical axial histidine is absent in C‐extension‐type HOs (Supplementary text in the Supporting Information), suggesting that these enzymes have evolved alternative catalytic mechanisms.

We hypothesized that H172 of *So*HmuZ, the proximal axial ligand for heme conserved in *Vc*HutZ and *Hp*HugZ, but lost in *Pdc*HOA (Figure 6B), may be the key to this puzzle. To test this, an H172A variant of *So*HmuZ was generated; clearly, the mutation nearly abolished hemin degradation activity (to 2%), and both binding affinity and cooperativity were undetectable (Table 1 and Figure 7A). MD simulations showed a dramatic increase in heme RMSD to ∼0.5–0.7 nm for the H172A variant (Figure 7E), and the heme was severely displaced within the pocket (Figure 7F and Movie S1 in Supporting Information). More importantly, this proximal instability propagated to the dimer interface, as the H172A mutation caused the most significant increase in the inter‐subunit distance (Figure 7D). Conversely, the H172A mutant retained 62% of its *V*_max_ and *k*_cat_ toward MP‐11, and its binding affinity was slightly enhanced (*K*_0.5_ remained at 79%), although cooperativity was markedly reduced (*n *= 1.21 vs. 2.05) (Table 1 and Figure 7A). Moreover, a triple mutant (L132A/D134L/H172A) designed to expand the active pocket was found to further boost MP‐11 degradation, yielding a twofold increase in catalytic rates (*V*_max_ and *k*_cat_ to 200%) and enhanced affinity (*K*_0.5_ decreased to 38%). Collectively, these data suggest a reciprocal stabilization mechanism: H172 strictly anchors the small‐molecule hemin for electron transfer, while the coordinated heme concomitantly reinforces the dimer interface. For MP‐11, however, its covalently attached peptide tail provides intrinsic steric bulk that likely allows the substrate to self‐stabilize and fix its proper orientation.

## DISCUSSION

The maintenance of heme homeostasis, which is pivotal for bacterial survival and proliferation, requires a tight balance between synthesis, utilization, and degradation^1^, ^62^. By degrading heme, HOs are established to manage heme toxicity, generate signaling molecules such as carbon monoxide and bilirubin, and facilitate the acquisition of heme from the surroundings to fulfill iron requirements^52^, ^63^. Characterized HOs are structurally diverse and capable of carrying out the oxidative cleavage of heme's porphyrin ring at varying positions. To date, all but one are known to degrade heme *b* exclusively^20^, ^64^. Given the existence of an HO that breaks down heme *c,* but not heme *b* ^1^, it is reasonable to propose that living organisms must have evolved HOs that can process both heme *b* and heme *c*.

In this study, such HOs were captured through a comprehensive pipeline spanning *in silico* analyses of gene distribution patterns, SSN/phylogeny, and active site architecture. We first looked into the heme uptake module, in which the genes for all types of HOs are commonly clustered on the chromosome. Despite this, the distribution and evolution of HOs are rather complex, presumably resulting from multiple ancestors, high susceptibility to mutations, and extensive horizontal transfer^6^, ^25^, ^26^. Active site architecture analysis clearly indicated that HOs of the Core‐only type are most likely to possess the potential to degrade both heme *b* and heme *c*. By combining the phylogenomics data, we were finally able to narrow down the scope for such HOs to *γ‐proteobacteria*, among which HmuZ of *S. oneidensis* was chosen for validation because the bacterium is particularly rich in cyt *c* proteins and encodes only one HO^49^, ^50^.

Indeed, *So*HmuZ shows degrading activity for both heme *b* and heme *c* (the latter from proteolytically processed cyt *c* proteins). Mechanistically, the dual‐substrate scope arises from a functional separation in substrate handling. The lineage‐specific histidine residue (H172 in the case of *So*HmuZ), conserved within these selected Core‐only‐ and N‐fusion‐type HOs, is essential for the precise coordination of heme *b* ^43^, ^47^, but becomes dispensable for bulkier heme *c*–peptide complexes^20^. This functional dichotomy reflects a “Core‐only/His + ” architecture: An open active site accommodates peptide‐bound heme *c*, while the histidine tether ensures efficient heme *b* turnover. As observed in characterized homologs (e.g., *Vc*HutZ), this structurally economical solution elegantly balances accessibility with specificity, allowing this class of HOs to maintain broad catalytic competence within a minimal framework. In the case of heme *c*, our data suggest that the interaction between bulky MP‐11 and the active pocket may facilitate the proper orientation of heme *c*, allowing the catalytic reaction to proceed.

In this study, we also made efforts to address why HOs of the N‐fusion and C‐extension types could not work with heme *c*. N‐fusion‐type HOs, such as *Hp*HugZ, fail because the extra domain likely acts as a steric barrier preventing access by the bulkier heme *c*. This notion gains support from the finding that *Hp*HugZ variants without this domain show enhanced heme *b* degradation activity^43^. A similar mechanism governed by extra domains likely accounts for the failure of HOs of the C‐extension type in degrading heme *c*. Interestingly, although C‐extension‐type HOs, similar to *Pdc*HOA, lack the conserved histidine, they are capable of degrading heme *b* ^48^. It has been proposed that these HOs use an extra functional domain called DRI to facilitate the degradation of heme *b* ^19^. DRI, which is found in the bacterial heme‐binding/utilizing proteins, uses another histidine residue as the secondary binding site to interact with heme *b* ^25^, ^52^.

Core‐only‐type HOs could work with heme *c*, with the caveat that cyt *c* proteins are proteolytically digested. This can be readily explained by the substantial size of intact cyt *c* proteins relative to the active pocket of the enzymes. The proteolytic process occurs in the periplasm, consistent with the earlier suggestion that periplasmic protease DegP of *E. coli* plays a major role in recycling cyt *c* proteins^65^. It should be noted that the proteolytic digestion must generate heme *c*–peptide complexes with stochastic variability with respect to length and sequence. This would be particularly true in *S. oneidensis*, which expresses over 40 cyt *c* proteins, implying that *So*HmuZ, and possibly all HOs that work with heme *c*, operate on a heterogeneous substrate pool^11^. Accordingly, we propose that substrate recognition is centered on the heme core rather than the surrounding peptide context. In line with this, the *in vitro* kinetic rates of *So*HmuZ for degrading MP‐11 appear to be relatively modest, a result primarily constrained by the inherent limitations of this extensively truncated surrogate, which fails to recapitulate the complex conformations of the native intermediates generated *in vivo*. Furthermore, our proposal perfectly aligns with the physiological context because the clearance of senescent or misfolded cyts *c* is intrinsically a continuous process that occurs at a steady state and does not necessitate exceptionally high instantaneous turnover rates characteristic of primary metabolic enzymes. More importantly, this modest catalytic efficiency likely represents a universal biochemical baseline for this class of enzymes when processing covalently peptide‐tethered substrates. Indeed, *Pdc*HOA shows a comparably low turnover rate of the exact same magnitude (*k*_*cat*_ = 0.076 min^−1^) when catalyzing MP‐11 *in vitro* ^20^. Such accommodation of molecular diversity constitutes a critical adaptive strategy, ensuring metabolic resilience during the stochastic turnover of intracellular cyt *c* proteins.

While existing biochemical and genetic evidence robustly supports the DegQ‐coupled “deconstruction‐first” model, the macroscopic phenotypes of mutants in a complex *in vivo* environment often show physiological pleiotropy. Specifically, the loss of DegQ not only impedes the generation of the heme recycling precursor pool but also triggers periplasmic quality control defects and outer‐membrane abnormalities^55^, ^56^, which likely exert synergistic toxicity with the electron transport perturbations caused by the abnormal aggregation of uncleaved hemoproteins, such as overproduced TsdB^66^. Consequently, the accumulation of cyts *c* and the observed growth defects in the Δ*degQ* mutant reflect a compound physiological stress driven jointly by an impaired recycling pathway and perturbed periplasmic hemoprotein homeostasis. Future systematic resolution of the precise *in vivo* metabolic flux of these heme–peptide intermediates will further close the mechanistic loop of this physiological pathway. Given that HOs, such as *Vc*HutZ^23^, are required for efficient utilization of heme as an iron source in general, we expect that iron released by this class of dual‐function HOs is subsequently reutilized^64^. Despite this, our data suggest that *So*HmuZ plays a larger role in cyt *c* quality control than in iron recycling, as the cyts *c* that are no longer needed appear more detrimental to the cell.

Ultimately, this work provides an integrated evolutionary, structural, biochemical, and physiological framework that defines a novel strategy for bacterial heme recycling. By successfully capturing the long‐missing dual‐function HOs that work with both heme *b* and heme *c*, we reveal that these universally distributed Core‐only HOs with an axial histidine ligand rely on a synergistic “deconstruction‐first” mechanism, serving as a vital functional interface between cyts *c* turnover and iron reclamation. This remarkable structural and biochemical plasticity ensures efficient maintenance of the intracellular heme pool, conferring a critical adaptive advantage that confers bacteria with the metabolic flexibility required to sustain cellular balance and iron homeostasis under fluctuating environmental pressures.

## MATERIALS AND METHODS

### Bacterial strains, plasmids, and culture conditions

The list of all bacterial strains and plasmids used in this study is shown in Table S2. The primers used in this study are listed in Table S3. Unless otherwise specified, chemicals were sourced from Sigma. For genetic editing purposes, *S. oneidensis* and *E. coli* strains were cultured under aerobic conditions in LB; Difco comprising 10 g/l tryptone, 5 g/l yeast extract, and 5 g/l NaCl, at 30°C and 37°C, respectively. When necessary, the growth medium was supplemented with chemicals at the following final concentrations: 2,6‐diaminopimelic acid (DAP) at 0.3 mM, kanamycin (Km) at 50 μg/ml, and gentamicin (Gm) at 15 μg/ml. Growth of *S. oneidensis* strains was monitored by measuring the optical density at 600 nm (OD_600_) in triplicate. For aerobic growth, 3 ml of fresh medium was inoculated with 30 μl of an overnight culture and incubated at 30°C with shaking at 250 rpm.

### In‐frame deletion mutagenesis and inducible gene expression

In‐frame deletion mutants of *S. oneidensis* were generated using an *att*‐based fusion PCR method as described previously^67^. The flanking regions of the target genes were amplified with primers containing *attB sites*, followed by secondary fusion PCR. The resulting constructs were cloned into the plasmid pHGM01 using the Gateway BP Clonase II enzyme mix (Invitrogen) and maintained in the DAP‐auxotrophic *E. coli* WM3064. The mutant constructs were introduced into *S. oneidensis* by conjugation. Colonies showing Gm sensitivity and sucrose resistance were selected as candidate deletion mutants. Deletion of the target gene was initially assessed by PCR and subsequently confirmed by DNA sequencing.

In order to complement the mutants and induce gene expression, the relevant genes amplified by PCR were cloned into pHGEN‐P*_tac_* under the control of the isopropyl β‐d‐1‐thiogalactoside (IPTG)‐inducible promoter P*_tac_* ^68^. Sequencing was performed to verify that the resulting plasmids were correctly constructed and transferred into the relevant strains via conjugation.

### Site‐directed mutagenesis

The *hmuZ* gene in pHGEN‐P*_tac_* was targeted for site‐directed mutagenesis using the QuikChange II XL kit (Agilent). Point mutations were introduced according to the manufacturer's instructions. The resultant mutant plasmids were digested with *Dpn*I for 6 h at 37°C, transformed into *E. coli* WM3064, and validated by sequencing. The confirmed plasmids were subsequently introduced into the *S. oneidensis* strains to examine the effects of the mutations.

### Recombinant protein expression and purification

The recombinant hexahistidine (His_6_)‐tagged *So*HmuZ, *Hp*HugZ, *Vc*HutZ, and site‐directed mutants of *So*HmuZ were cloned into the expression vector pET28A and expressed in *E. coli* BL21 (DE3). For *So*HmuZ, the His_6_ tag was placed at the C terminus to preserve the open N‐terminal entry path of the Core‐only architecture and to prevent the introduction of an artificial N‐terminal extension that could interfere with access of peptide‐tethered heme *c*. His_6_‐tagged ScyA and TsdB from pHGEN‐P*_tac_* were expressed and purified from *S. oneidensis*. Protein expression was induced with 0.5 mM IPTG at 16°C for 16 h. Cells obtained from 2 l cultures were collected by centrifugation at 2500*g* for 20 min and washed twice with ice‐cold binding buffer containing 20 mM Tris‐HCl and 150 mM NaCl at pH 7.0. The resulting cell pellets were resuspended in 40 ml of the same buffer and disrupted by sonication. Insoluble cell debris was then removed by centrifugation. The clarified lysate was then applied to a nickel chelate column (Qiagen), washed with 20 mM imidazole, and the proteins were eluted with 150 mM imidazole. Proteins were further concentrated using ultrafiltration and dialyzed into 20 mM Tris‐HCl (pH 7.0) with 150 mM NaCl. Protein concentration was determined using the Bradford assay (Bio‐Rad) with bovine serum albumin as the standard.

### SDS‐PAGE, Western blotting, and heme staining

Cells at the mid‐log phase were harvested, washed with Tris‐HCl buffer (pH 7.0), and lysed by sonication on ice. Lysates were mixed with 4× SDS loading buffer, boiled at 100°C for 10 min, and separated by SDS‐PAGE (10% resolving, 5% stacking gels). Proteins were visualized by Coomassie Brilliant Blue R‐250 staining, heme staining using 3,3′,5,5′‐tetramethylbenzidine, or Western blotting^69^.

Proteins were transferred to PVDF membranes, blocked with TBST containing 5% BSA, and probed with a mouse monoclonal His‐tag antibody (1:5000), followed by HRP‐conjugated goat anti‐mouse IgG (1:10,000). Signals were detected using chemiluminescence and imaged with a ChemiScope 6000 system.

### Heme *c* assays

Cultures of *S. oneidensis* strains were grown to the early stationary phase, harvested by centrifugation, and cell pellets were collected. The abundance of heme *c* was initially estimated from pellet coloration. Total cellular proteins were extracted using a bacterial protein extraction kit (Sangon Biotech). For heme *c* quantification, protein extracts were precipitated with trichloroacetic acid (TCA) to remove non‐covalently bound heme, and the resulting protein pellet (containing covalently bound heme *c*) was used for analysis^70^. Equal volumes of cell suspensions (50 μl) were analyzed using a QuantiChrom heme assay kit (BioAssay Systems) according to the manufacturer's instructions. Heme *c* levels were normalized to the total protein content and expressed relative to the WT.

### Heme binding and affinity assays

The binding stoichiometry between *So*HmuZ and hemin or MP‐11 was determined by UV–visible spectroscopic titration^71^. Assays were performed with 10 μM *So*HmuZ in the presence of 20 μM BSA (for hemin titration) to minimize nonspecific binding, in 20 mM Tris‐HCl and 150 mM NaCl (pH 7.0), with buffer alone as a control. Incremental addition of the substrate resulted in a progressive increase in the Soret band (at 420 nm for the hemin complex and 408 nm for the MP‐11 complex), reaching saturation at an approximate 1:1 stoichiometry.

The dissociation constant (*K*_d_) and binding stoichiometry *N* were further determined by tryptophan fluorescence quenching. Hemin was titrated into an *So*HmuZ solution (20 mM Tris‐HCl and 150 mM NaCl, pH 7.0) and incubated for 10 min at 30°C or 37°C prior to measurement. Fluorescence emission spectra (300–500 nm) were recorded at an excitation wavelength of 295 nm using a TECAN Infinite M200 PRO microplate reader. *K*_d_ and *n* were calculated by fitting the Stern–Volmer equation.

$$\mathrm{lg}\frac{F_{0}-F}{F}=-\mathrm{lg}K_{d}+N\mathrm{lg}\left[Q\right]$$

*F*_0_ and *F* represent the fluorescence intensities of *So*HmuZ in the absence and presence of hemin, respectively; [*Q*] denotes the hemin concentration (mol/l); *K*_d_ is the dissociation constant (μM); and *N* is the number of binding sites^18^.

### Heme oxygenase activity assays

The heme oxygenase activity of *So*HmuZ toward heme *b* was evaluated spectrophotometrically^72^. To prepare the reactant complex, hemin was incrementally added in 2 µM increments (up to a total of 20 µM) to a final concentration of 10 µM *So*HmuZ. This mixture was prepared in a reaction buffer containing 20 mM Tris‐HCl, 150 mM NaCl (pH 7.0), 20 µM BSA, and 2 µM catalase at room temperature, yielding a final volume of 200 µl. The catalytic turnover was initiated by the addition of 10 mM ascorbic acid, and UV–visible absorption spectra (300–800 nm) were recorded at 1‐min intervals over a 5‐min duration using a TECAN Infinite M200 PRO microplate reader.

For assays involving heme *c*, intact equine heart cyt *c* (≥95%; Sigma‐Aldrich), TsdB, and ScyA were first subjected to proteolytic cleavage by incubation with trypsin at a 10:1 (w/w) protein‐to‐protease ratio at 37°C for 1 h to generate shorter heme *c* peptides. The digestion was terminated by adding 1 mM phenylmethylsulfonyl fluoride (PMSF)^73^. Subsequently, full‐length equine heart cyt *c*, TsdB, ScyA, and their trypsin‐digested products, including microperoxidase‐11 (MP‐11; Sigma‐Aldrich), were each diluted to a final concentration of 10 µM and incubated with 10 µM HOs. These reactions were carried out in 20 mM Tris‐HCl and 150 mM NaCl (pH 7.0) supplemented with 20 µM BSA and 2 µM catalase at room temperature, with a total volume of 200 µl per well in 96‐well microtiter plates. Similar to the heme *b* assay, the reaction was initiated via the addition of 10 mM ascorbic acid, and spectra (300–800 nm) were recorded every minute for 5 min. Negative controls were established by substituting the HOs with an equal volume of reaction buffer under otherwise identical conditions.

### Steady‐state kinetic analysis

Steady‐state kinetic assays were conducted to evaluate the kinetic properties of *So*HmuZ variants^20^. Triplicate reactions were carried out in a final volume of 200 μl containing 20 mM Tris‐HCl and 150 mM NaCl (pH 7.0) in 96‐well plates. Each reaction mixture contained a fixed total enzyme concentration ([*E*]_*t*_) of 0.5 μM, 10 mM sodium ascorbate to initiate and sustain the reaction, and varying concentrations of substrate (1–6 μM for hemin; 1–5 μM for MP‐11). The degradation of the substrates was monitored continuously by measuring the decrease in absorbance at 412 nm (hemin) or 408 nm (MP‐11) using a microplate reader. Initial reaction rates ($v_{0}$) were extracted from the linear portion of the progress curves and plotted against the respective substrate concentrations ([*S*]), and kinetic parameters were determined by non‐linear regression fitting to the Hill equation using GraphPad Prism 9.0:

$$v_{0}=\frac{V_{\max}{\left[S\right]}^{n}}{K_{0.5}^{n}+{\left[S\right]}^{n}}$$

This non‐linear regression yielded the maximum reaction velocity (*V*_max_), the apparent half‐saturation constant (*K*_0.5_), and the Hill coefficient (*n*). Furthermore, the catalytic turnover number (*k*_cat_) for each variant was calculated as *V*_max_/[*E*]_*t*_.

### LC‐MS/MS analysis

Reaction products from the *So*HmuZ‐catalyzed degradation of MP‐11 were analyzed by LC–MS/MS. Samples were quenched with cold methanol, centrifuged (12,000*g*, 10 min, 4°C), and filtered through a 0.22 μm membrane. Chromatographic separation was performed on an ACQUITY UPLC BEH C18 column (2.1 × 100 mm, 1.7 μm particle size; Waters) maintained at 30°C. The mobile phase consisted of water with 0.1% formic acid (solvent A) and acetonitrile with 0.1% formic acid (solvent B). Elution was carried out using a linear gradient of 5%–95% solvent B over 10 min at a flow rate of 10 μl/min, using an LC‐400 micro‐flow liquid chromatography system (SCIEX). Mass spectrometric analysis was conducted on a TripleTOF 6600 high‐resolution mass spectrometer (SCIEX) equipped with an electrospray ionization (ESI) source operating in positive‐ion mode. Full MS scans were acquired over an *m/z* range of 100–2000, followed by data‐dependent MS/MS (dd‐MS2) fragmentation of selected precursor ions using collision‐induced dissociation (CID; 20–40 eV). Charge states were assigned based on isotopic patterns, and chromatographic peaks were correlated with mass spectra by retention time.

### Phylogenetic analysis

An SSN comprising 16,400 sequences from the HugZ‐like (IPR037119) and HutZ (IPR014419) families was constructed via the EFI‐EST web server^44^, ^45^ (*E*‐value threshold 10^−5^; ∼30% identity). The network was then filtered with an alignment score threshold of 40–290 and visualized in Cytoscape v3.10.4 using the yFiles organic layout^74^. From individual SSN clusters, 339 representative sequences were curated for phylogenetic analysis. Using NCBI Batch CD‐Search^75^ (*E*‐value < 0.01) and a custom R pipeline^76^, sequences were truncated to retain only the homologous split β‐barrel core (TIGR04109 or PF13883), removing auxiliary domains. The extracted core sequences were aligned using MAFFT^77^ (L‐INS‐i strategy; >50% gap filtration). A neighbor‐joining tree was constructed in MEGA 11^78^ (Poisson correction, 1000 bootstraps), visualized using Chiplot^79^, and annotated with domain architectures (N‐fusion, C‐extension, or Core‐only) and distal axial histidine conservation (His+/His−).

### MD simulations

Initial *So*HmuZ homodimer–heme *b* complex models (WT, R94A, H172A) were generated using AlphaFold 3^57^ and prepared using the CHARMM‐GUI Solution Builder^80^. Systems were parameterized with the CHARMM36m force field^81^ and TIP3P water, solvated in a cubic box (with a 1.0 nm margin), and neutralized and adjusted to an ionic strength of 0.15 M NaCl. Simulations were conducted in GROMACS 2024.4^82^. Following steepest‐descent energy minimization, systems were equilibrated using a two‐step protocol: a 100 ps NVT phase (303.15 K, V‐rescale thermostat) and 1 ns NPT equilibration at 1 bar using the Berendsen barostat, with positional restraints applied to heavy atoms. Production MD was performed for 100 ns in the NPT ensemble using the Parrinello–Rahman barostat. Simulation parameters included PME electrostatics (1.2 nm cutoff) and van der Waals interactions treated with a force‐switching function (1.0–1.2 nm). All bonds involving hydrogen atoms were constrained using the LINCS algorithm, allowing for a 2 fs integration time step. Trajectories were analyzed using GROMACS built‐in tools and the MD Analysis library^83^ to extract the root‐mean‐square deviation (RMSD) of the heme, the distances between R94 and the heme propionate groups, the number of water molecules within 5 Å of the active site, and dimer interface metrics. Structural visualizations were rendered using PyMOL.

### Statistics and reproducibility

All experiments were performed in independent triplicates, and data are presented as mean ± SD. Statistical significance was evaluated using one‐way ANOVA, followed by Tukey's post hoc test for multiple comparisons (*p < 0.05, **p < 0.01, and ***p < 0.001).

## AUTHOR CONTRIBUTIONS

**Jiyuan Hou**: Data curation; formal analysis; investigation; validation; writing—original draft. **Wenhan Dong**: Investigation. **Hui Yang**: Investigation. **Yuxuan Liang**: Investigation. **Haichun Gao**: Conceptualization; funding acquisition; project administration; supervision; writing—review and editing.

## ETHICS STATEMENT

This article does not contain any studies with human participants or animals performed by any of the authors.

## CONFLICT OF INTERESTS

The authors declare no conflict of interests.

## Supporting information

Supporting File 1.

Supporting File 2.

## Data Availability

The data that support the findings of this study are available within the paper and its Supporting Information. The datasets for the MD simulations are available at Zenodo (https://doi.org/10.5281/zenodo.19049724).
