# Supplementary material for: Capture of long‐missing heme oxygenases that degrade both heme b and heme c
Source: mLife. 2026 Oct 28;5(5):552–67. doi: 10.1002/mlf2.70118 (PMC13715372; doi:10.1002/mlf2.70118)
Supplement: Supplementary file 1 [file MLF2-5-552-s002.pdf]

Supporting Information

Capture of long-missing heme oxygenases that degrade both heme *b* and heme *c*

Jiyuan Hou[1,2], Wenhan Dong[1], Hui Yang[1], Yuxuan Liang[1,2], and Haichun Gao[1,2*]

[1]Institute of Microbiology and [2]State Key Laboratory for Vegetation Structure Function and Construction (VegLab), College of Life Sciences, Zhejiang University, Hangzhou, 310058, China

*** Correspondence:** Haichun Gao, haichung@zju.edu.cn

Institute of Microbiology and College of Life Sciences, Zhejiang University, Hangzhou, Zhejiang, 310058, China

Tel. & Fax (+86) 571 88981107

The Supporting Information includes:

Figure S1-S10

Supplementary Text
Structural and functional divergence of the C-extension lineage

Table S1-S3

Movie S1 (provided as an MP4 file)
Molecular dynamics (MD) simulation of *So*HmuZ. This movie shows the MD trajectories, illustrating the pronounced displacement of the heme group within the binding pocket in the H172A mutant (orange) relative to the WT (grey) and R94A variant (blue). The protein backbones are shown in transparent cartoon representation, and the heme molecules are shown as sticks color-coded by their respective variants.

Supporting Information References

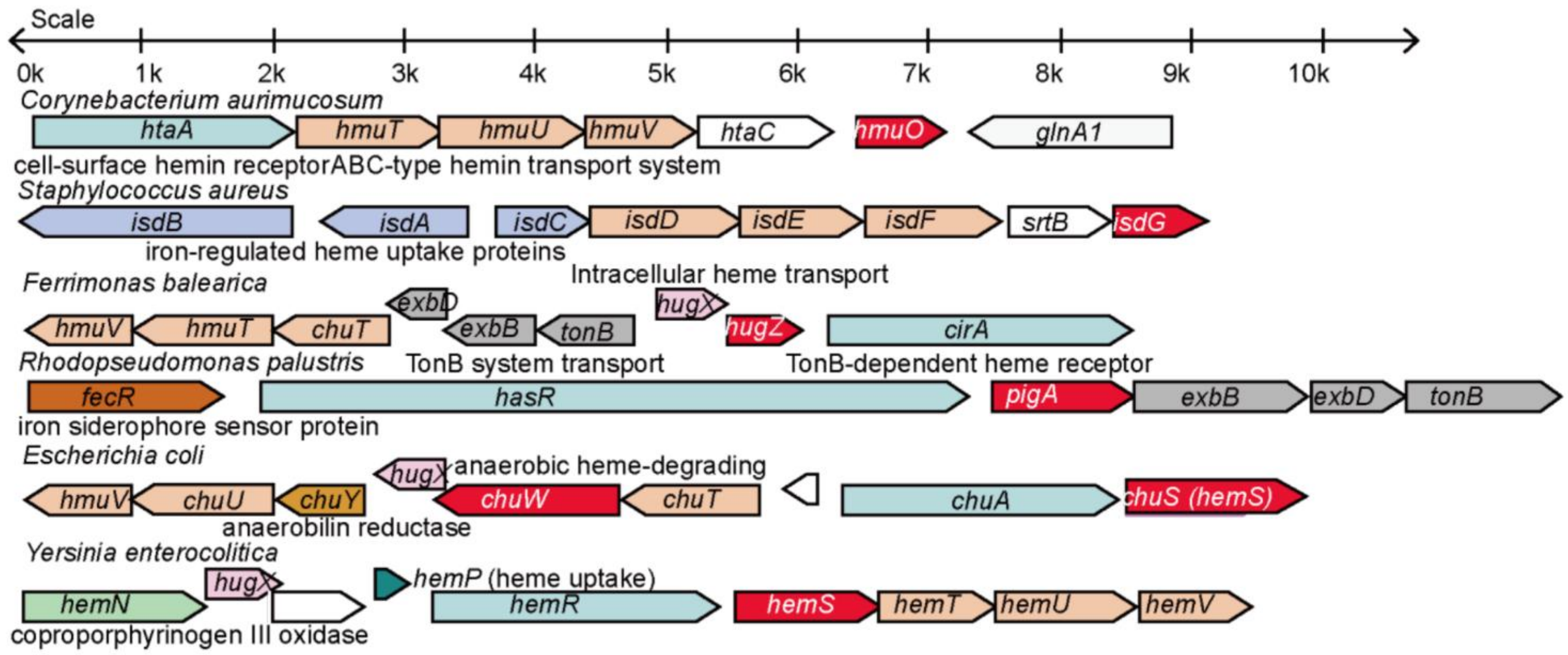

Supplementary Figure 1. Genomic organization of heme uptake and degradation clusters. Representative genomic neighborhoods illustrate the conserved synteny between heme oxygenase genes (red) and heme uptake systems across diverse bacterial species, suggesting a tight functional coupling between environmental heme acquisition and intracellular degradation.

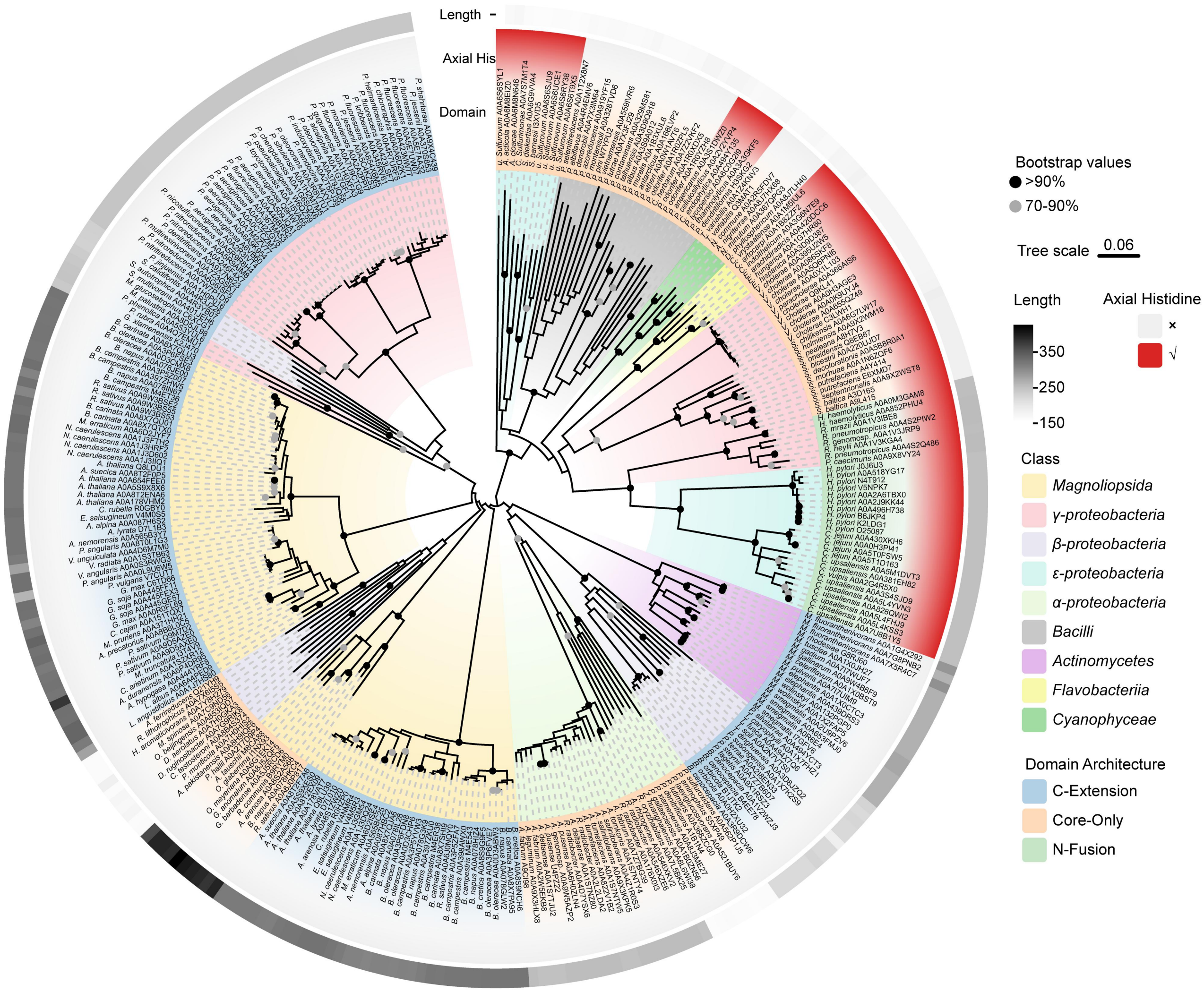

Supplementary Figure 2. High-resolution phylogenetic tree of the HugZ-type heme oxygenase superfamily based on core domain sequences. This is the fully expanded and detailed version of the Neighbor-Joining (NJ) tree presented in Figure 3. The tree was constructed using 339 core domain sequences aligned by MAFFT. Terminal branches (leaves) are explicitly labeled with their corresponding taxonomic species names and UniProt accession IDs. As in Figure 3, the concentric rings annotate taxonomic lineages via branch background colors, structural domain architectures (C-Extension/blue, Core-Only/orange, N-Fusion/green), sequence lengths via a grayscale gradient (~150 to 450 aa), and the conserved axial histidine ligand (red for present, white for absent).

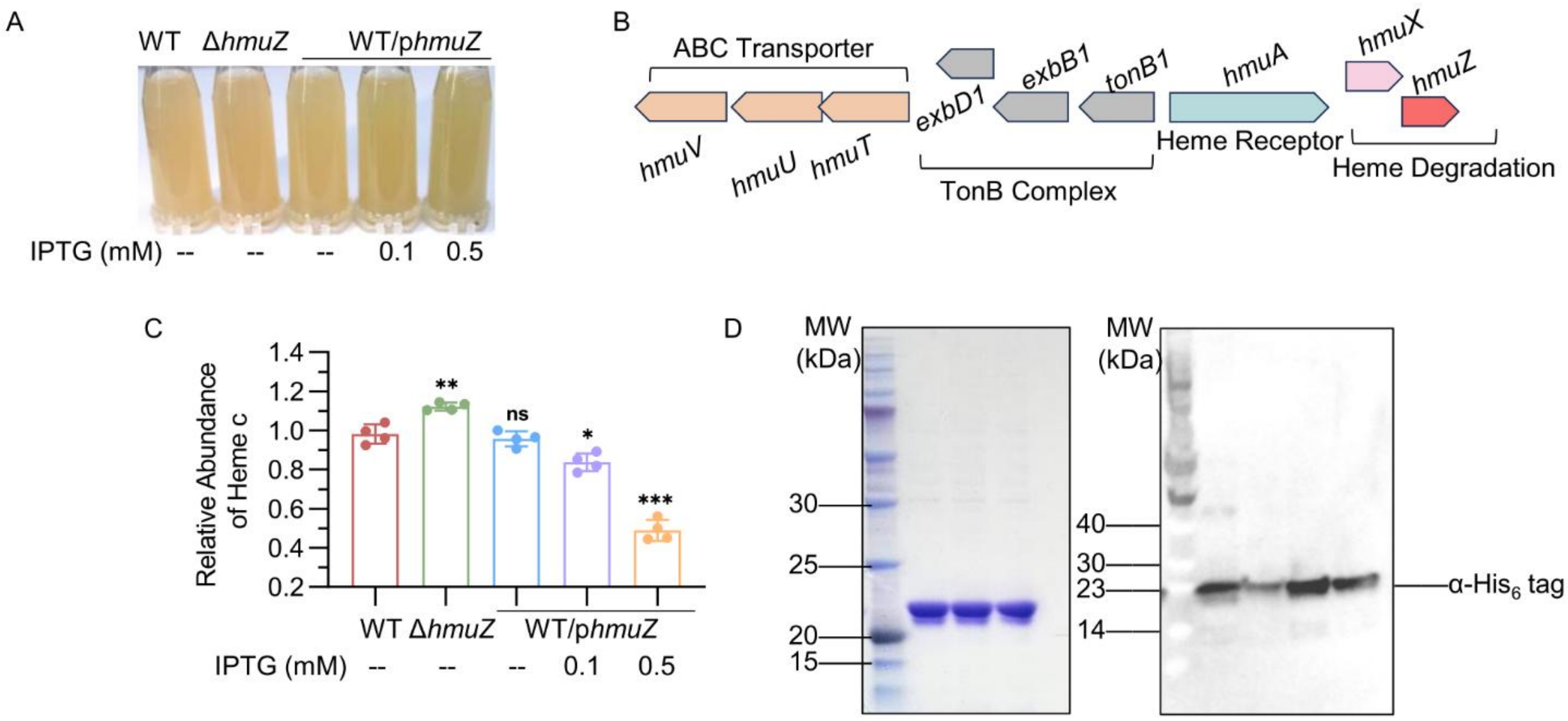

Supplementary Figure 3. Genomic context of *hmuZ* and its impact on cellular heme *c* levels. (A) Macroscopic appearance of liquid cultures of *S. oneidensis* WT, Δ*hmuZ*, and WT/p*hmuZ*. (B) Genetic organization of the *hmu* gene cluster in *S. oneidensis*. (C) Quantification of relative intracellular heme *c* abundance in the indicated strains. Data were normalized to total protein concentration and expressed relative to the WT. Error bars represent mean ± SD (n = 4). Statistical significance was determined by one-way ANOVA compared to the WT group (* $p < 0.05$, ** $p < 0.01$, *** $p < 0.001$; ns, not significant). (D) SDS-PAGE (left) and Western blot (right) analysis of the purified *So*HmuZ protein.

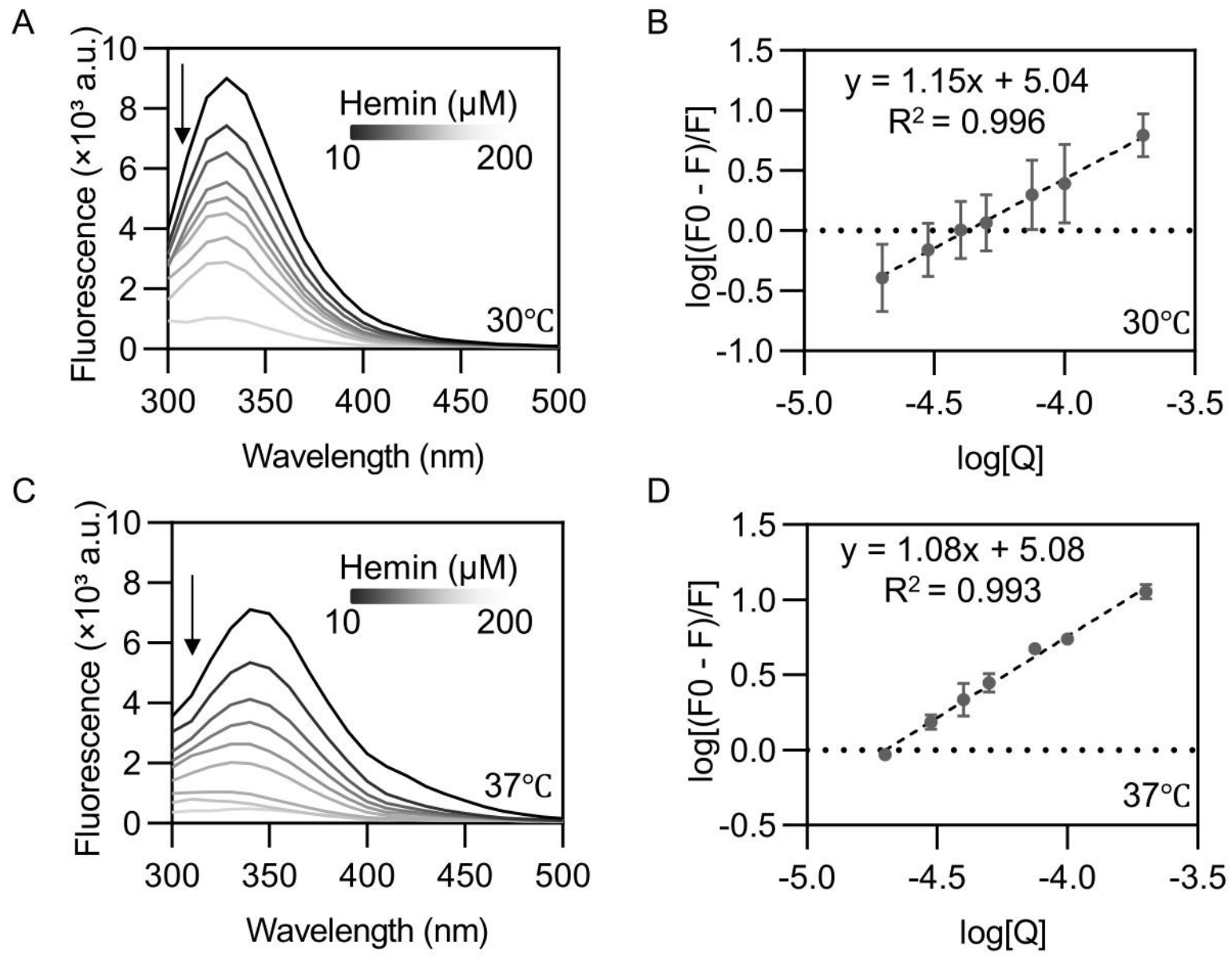

Supplementary Figure 4. Fluorescence quenching analysis of the interaction between *So*HmuZ and hemin. (A, C) Fluorescence emission spectra of *So*HmuZ (recorded at λ_{ex} = 295 nm) in the presence of increasing concentrations of hemin (10 – 200 μM at 30 °C (A) and 37 °C (C). The downward arrows indicate the concentration-dependent quenching of the intrinsic protein fluorescence. (B, D) Linear regression analysis using the Stern-Volmer equation at 30 °C (B) and 37 °C (D). Data points and error bars represent the mean ± standard deviation (SD) derived from three independent experimental replicates. The dashed lines show the linear fit applied to the mean values. The determined slopes (n≈1) and high goodness-of-fit (R^2> 0.99) confirm a 1:1 binding stoichiometry. Detailed binding parameters, including calculated K_d values, are provided in Table S1.

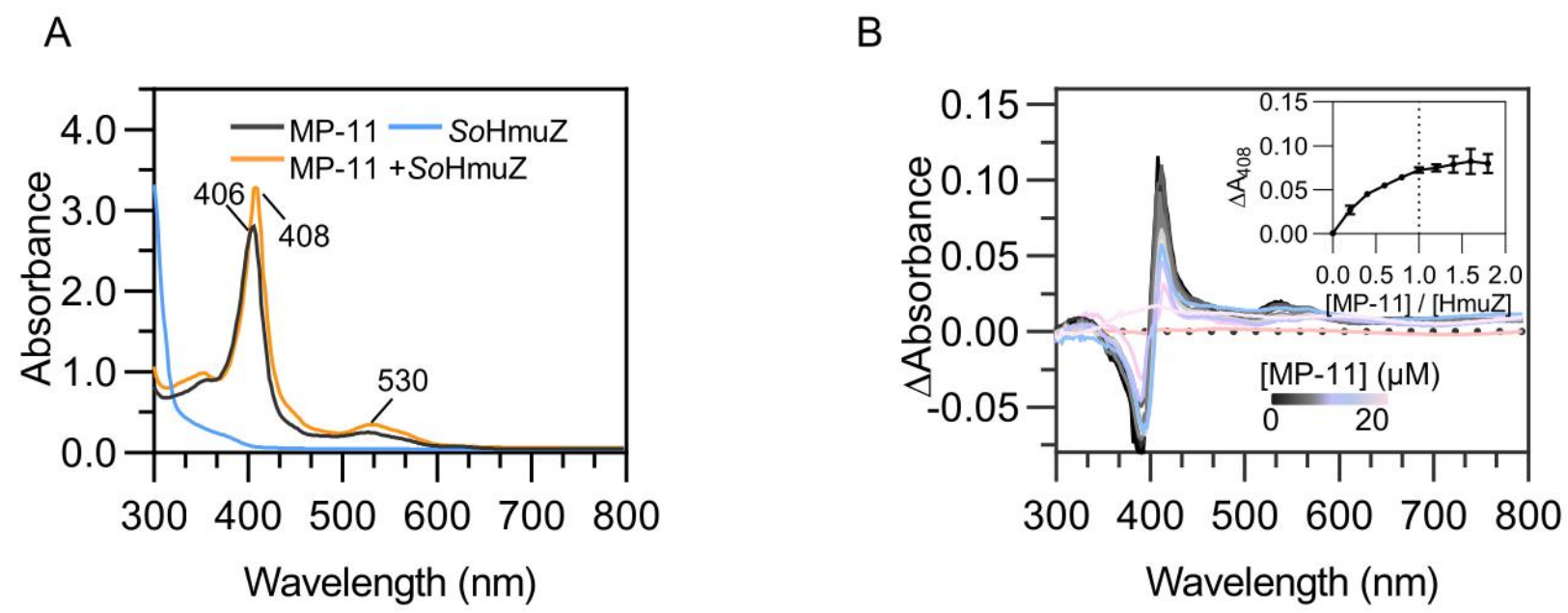

Supplementary Figure 5. Spectroscopic characterization of MP-11 binding to *So*HmuZ. (A) UV-visible absorption spectra of free MP-11 (black line), apo-*So*HmuZ (blue line), and the *So*HmuZ–MP-11 complex (orange line). Upon binding, the Soret band of MP-11 shifts from ~406 nm to 408 nm. (B) MP-11 binding activity of *So*HmuZ monitored by differential absorption spectroscopy. Spectral changes are shown during the titration of apo-*So*HmuZ with increasing concentrations of MP-11 (0–20 μM) in 20 mM Tris-HCl, 150 mM NaCl (pH 7.0). Inset: The corresponding titration curve plotted as the change in absorbance at 408 nm (ΔA_{408}) versus the [MP-11] / [*So*HmuZ] molar ratio. Error bars represent standard deviations (SD) from three independent replicates (n = 3).

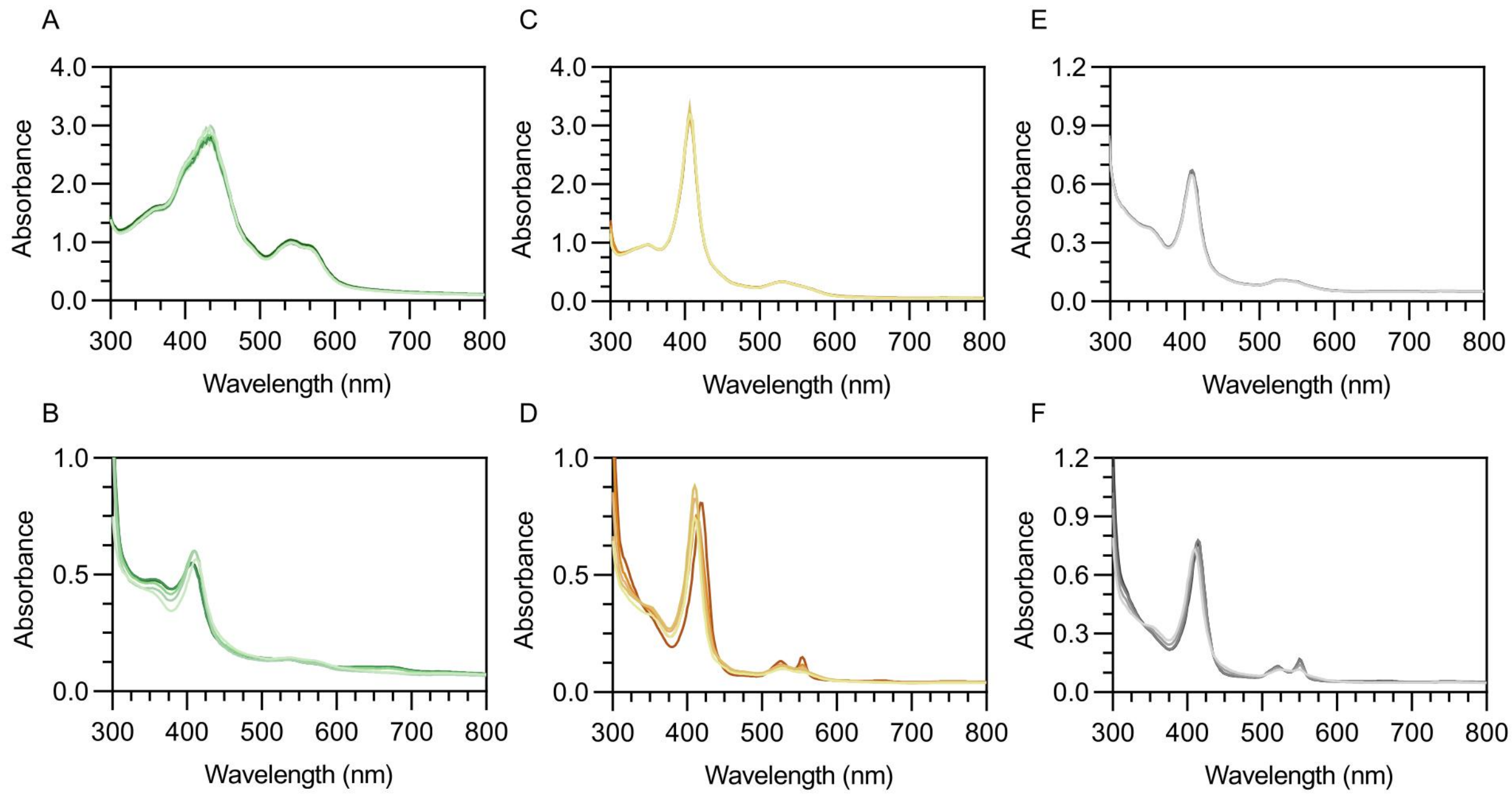

Supplementary Figure 6. Negative control assays for hemin, MP-11, and equine heart cyt *c* degradation. UV-vis spectral changes of hemin (A, B), MP-11 (C,D), and equine heart cyt *c* (E,F) were monitored under specific control conditions. Panels (A,C,E) show reactions containing *So*HmuZ but lacking the electron donor ascorbate. Panels (B,D,F) show reactions containing ascorbate but lacking *So*HmuZ. Assays were performed with 2 μM catalase and 20 μM BSA in 20 mM Tris-HCl, 150 mM NaCl (pH 7.0).

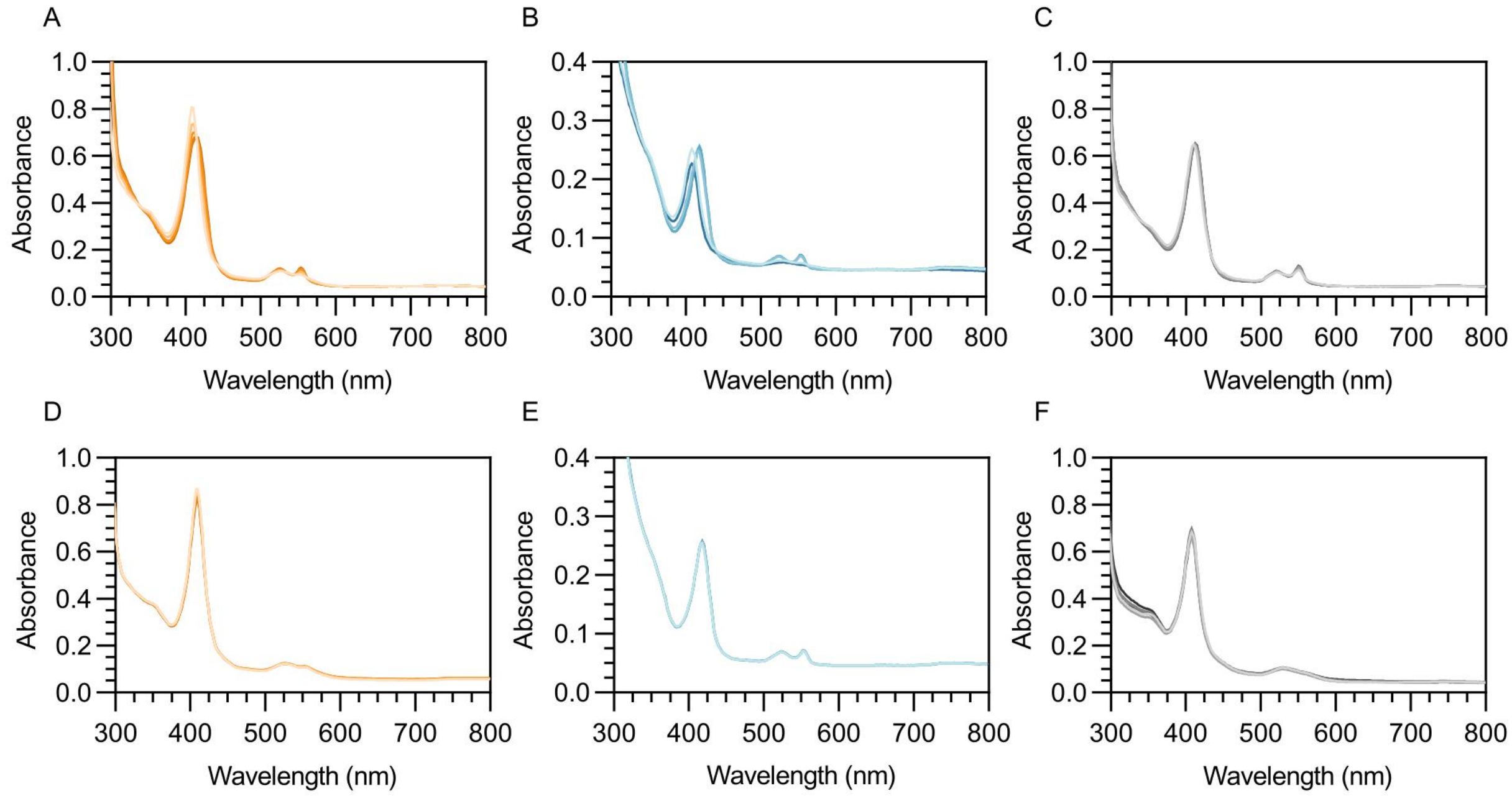

Supplementary Figure 7. Negative control assays for the degradation of trypsin-pretreated ScyA, TsdB, and equine heart cyt *c*. UV-vis spectral changes of trypsin-pretreated ScyA (A, D), TsdB (B, E), and equine heart cyt *c* (C, F) were monitored under specific control conditions. Panels (A–C) show reactions containing *So*HmuZ but lacking the electron donor ascorbate. Panels (D–F) show reactions containing ascorbate but lacking *So*HmuZ. Assays were performed with 2 μM catalase and 20 μM BSA in 20 mM Tris-HCl, 150 mM NaCl (pH 7.0).

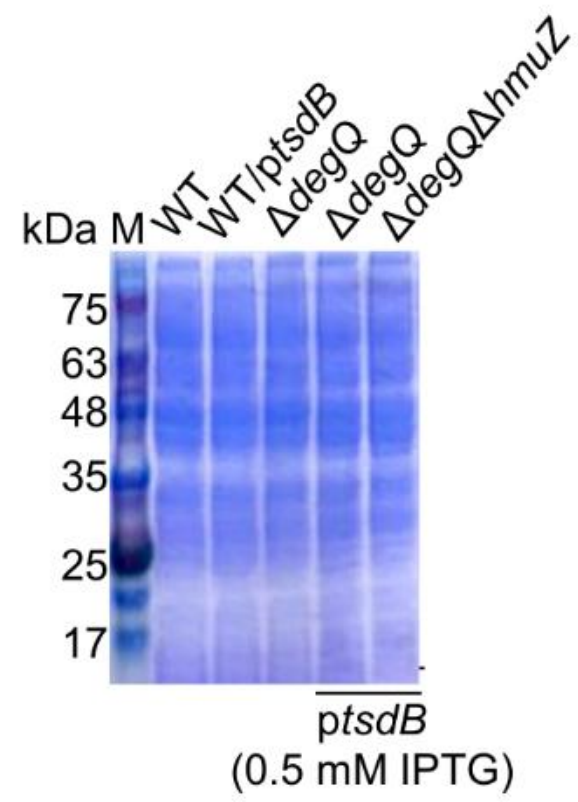

Supplementary Figure 8. Total protein loading control for the heme-staining analysis shown in Figure 5H. Coomassie blue-stained SDS-PAGE of total proteins from WT and the indicated mutant strains expressing TsdB. Cells were induced with IPTG as indicated. This analysis confirms equivalent total protein loading across all lanes, supporting the specific accumulation of heme *c* observed in the Δ*degQ* strain.

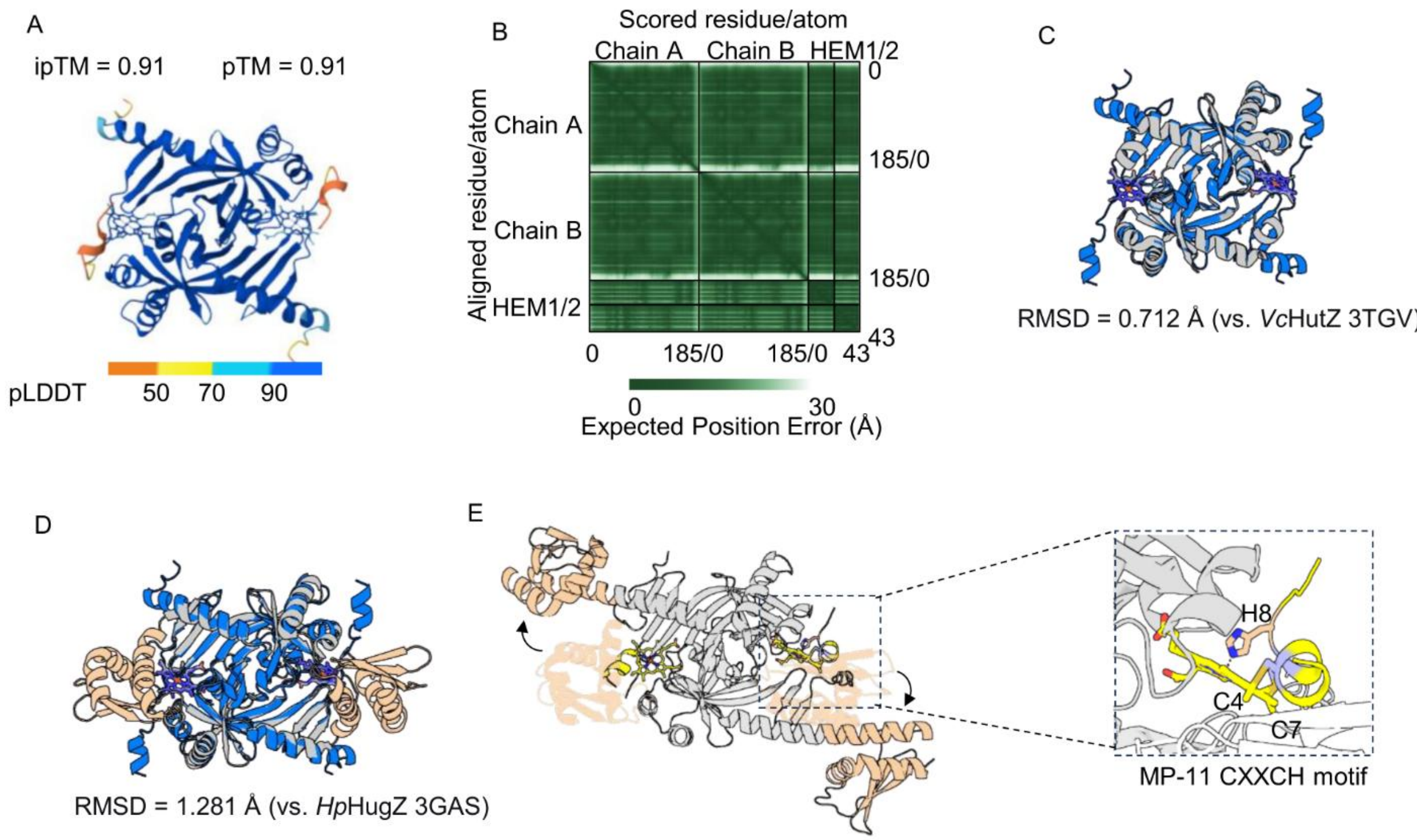

Supplementary Figure 9. Structural modeling and comparative analysis of *So*HmuZ and its homologs. (A) AlphaFold 3 predicted structure of the *So*HmuZ homodimer-hemin complex, colored according to the per-residue confidence score (pLDDT). The overall ipTM and pTM scores are indicated. (B) The corresponding Predicted Aligned Error (PAE) plot for the *So*HmuZ homodimer-hemin complex. (C, D) Structural alignment of the *So*HmuZ model (blue) with the crystal structures of (C) *Vc*HutZ (PDB: 3TGV, grey; RMSD = 0.712 Å) and (D) *Hp*HugZ (PDB: 3GAS; RMSD = 1.281 Å). For *Hp*HugZ, the core domain is colored grey, while the N-fusion domain is highlighted in wheat. (E) AlphaFold 3 predicted structure of the *Hp*HugZ homodimer in complex with MP-11. The structural overlay illustrates the lid-like opening and closing dynamics of the N-fusion domain (indicated by black arrows) upon binding. The inset highlights a magnified view of the MP-11 CXXCH motif within the binding pocket.

Supplementary Text: Structural and functional divergence of the C-extension lineage

Preliminary superposition of the Core-only type HOs representative *So*HmuZ and *Vc*HutZ, and the N-fusion type *Hp*HugZ shows that all these three HOs retain a proximal histidine (His172 in *So*HmuZ) as a conserved rigid axial ligand for heme (Figure 6A-B). In contrast, this critical residue is substituted by a water-mediated coordination network in HOZ (*Arabidopsis thaliana*), the representative of the C-extension type HOs (Figure. S10A).

Atomic-level inspection of the HOZ active site (PDB: 6M0A) shows that this water molecule is stabilized by a specific hydrogen-bonding network involving the amide groups of Gly212, Ala214, and Thr215 (Figure. S10B). This coordination shift directly results in a significantly diminished heme *b* affinity (K_d = 11±2 μM) and a loss of efficient catalytic activity[1]. Given that the *Arabidopsis* genome encodes several robust classical HO1-type HOs, these structural features collectively suggest that HOZ functions as an auxiliary regulator or molecular chaperone rather than a primary catabolic oxygenase[2].

Our phylogenetic analysis further supports these structural observations, revealing that the absence of the essential axial histidine is conserved across the entire C-extension type HOs (Figure. 3 and S2), indicating lineage-wide functional specialization. For instance, the GluTR-binding protein (GBP, Q9LU39), which resides within the same major evolutionary lineage as HOZ, has evolved into an auxiliary factor for regulating δ-aminolevulinic acid (ALA) synthesis[3]. This transition from catabolism to metabolic regulation is also evident in other C-extension type HOs, such as MSMEG_6519 (PDB: 5BNC), where the C-terminal DRI domain provides the structural flexibility required to integrate diverse ligand signals[4].

Notably, while *Pdc*HOA similarly lacks a proximal histidine, it represents a unique evolutionary branch of the Core-only type HOs. It utilizes the hydrophobic and steric properties of its covalently attached peptide tail to orient the substrate for regioselective cleavage, thereby enabling specialized heme *c* degradation. This mechanism is reinforced by its genomic proximity to the oligopeptide permease (Opp) system[5]. Based on these findings, we excluded the C-extension type HOs from subsequent kinetic analyses. This allowed our biochemical assessment to focus on representative members with high-turnover potential in the Core-only and N-fusion type HOs.

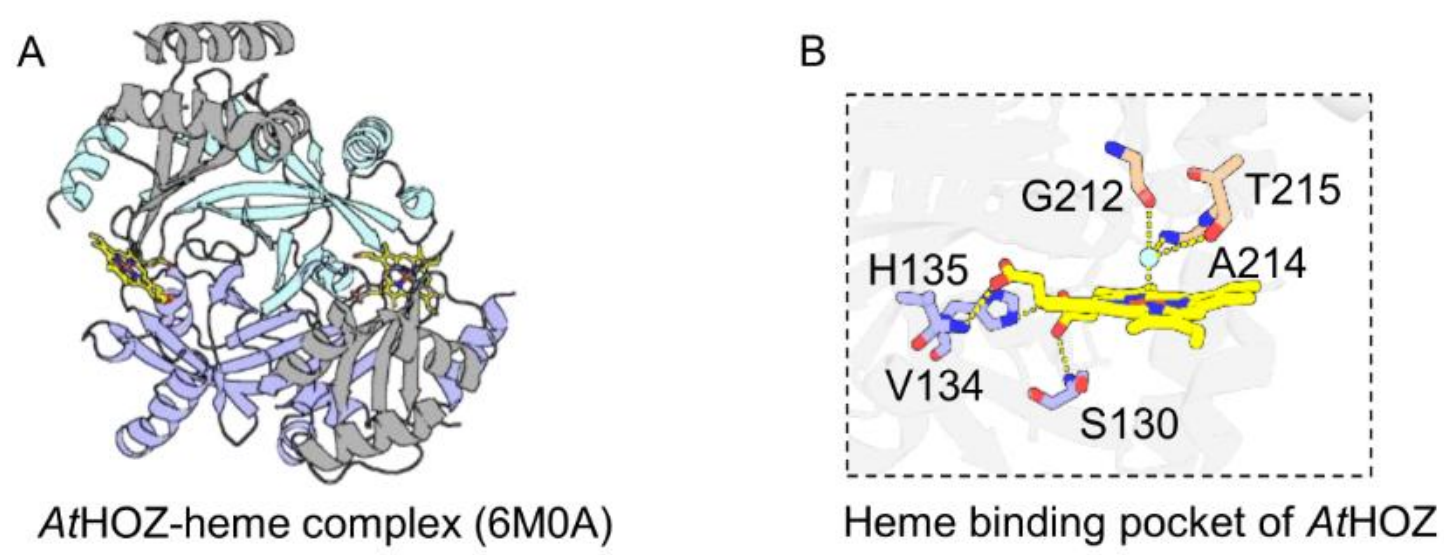

Supplementary Figure 10. Overall structure and water-mediated axial coordination of *At*HOZ. (A) Overall 3D structure of the *At*HOZ homodimer (PDB: 6M0A). The core domains of the two monomers are colored light blue and cyan, respectively, while their characteristic C-extension domains are highlighted in grey. (B) Atomic-level view of the heme-binding pocket. The classical proximal histidine is replaced by a water molecule (cyan sphere) that axially coordinates the heme iron. This structural water is stabilized by a hydrogen-bonding network involving residues Gly212, Ala214, and Thr215.

Table S1. Summary of binding parameters obtained from fluorescence quenching experiments.

Temperature	Replicate	Intercept (log K_d)	Slope/ Stoichiometry (n)	Goodness of Fit (R^2)	Calculated K_d (μM)[a]
30°C	#1	5.16	1.12	0.98	6.9
	#2	5.04	1.16	0.98	9.1
	#3	4.95	1.18	0.97	11.2
	Mean ± SD	—	1.15 ± 0.04	—	9.1 ± 2.2
37°C	#1	5.08	1.07	0.98	8.3
	#2	5.14	1.1	0.98	7.2
	#3	5.02	1.07	0.99	9.5
	Mean ± SD	—	1.08 ± 0.02	—	8.4 ± 1.2

[a] The apparent dissociation constant (K_d) was calculated via the equation $K_d = 10^{-\text{Intercept}}$.

Table S2. Strains and plasmids used in this study.

**Strain or plasmid**	**Description**	**Source/Reference**
E. coli strains		
DH5α	Host strain for plasmids	Laboratory stock
WM3064	Δ*dapA,* donor strain for conjugation	W. Metcalf, UIUC
BL21 (DE3)	Recombinant protein expression host strain	Laboratory stock
S. oneidensis strains		
MR-1	Wild type	Laboratory stock
HG3667	Δ*hmuZ* derived from MR-1	This study
HG3942	Δ*degQ* derived from MR-1	Laboratory stock
Plasmids		
pHGM01	Att-based suicide vector, Ap^r, Gm^r, Cm^r	6
pHGEN-Ptac	Gm^r, IPTG-inducible expression vecto	7
pHGEN-Ptac	Km^r, IPTG-inducible expression vecto	7
pET-28a(+)	His-tagged protein expression vector, Ap^r,Km^r	Novagen
pHGEN-P*tac-hmuZ*	Vector for inducible expression of His_6-tagged *hmuZ*	This study
pET-28a(+)-*hmuZ*	pET-28a(+) expressing His_6-tagged *hmuZ*	This study
pHGEN-P*tac-hmuZ*$^{(T29V)}$	Vector for inducible expression of His_6-tagged *hmuZ*$^{(T29V)}$	This study
pHGEN-P*tac-hmuZ*$^{(R94A)}$	Vector for inducible expression of His_6-tagged *hmuZ*$^{(R94A)}$	This study
pHGEN-P*tac-hmuZ*$^{(H172A)}$	Vector for inducible expression of His_6-tagged *hmuZ*$^{(H172A)}$	This study
pHGEN-P*tac-hmuZ* $^{(L132A/D134L/H172A)}$	Vector for inducible expression of His_6-tagged *hmuZ*$^{(L132A/D134L/H172A)}$	This study
pHGEN-P*tac-hutZ*	Vector for inducible expression of His_6-tagged *hutZ*	This study
pHGEN-P*tac-hugZ*	Vector for inducible expression of His_6-tagged *hugZ*	This study
pHGEN-P*tac-c*HOA	Vector for inducible expression of His_6-tagged *c*HOA	This study
pHGEN-P*tac-tsdB*	Vector for inducible expression of His_6-tagged *tsdB*	Laboratory stock
pHGEN-P*tac-degQ*	Vector for inducible expression of His_6-tagged *degQ*	This study
pHGEN-P*tac-scyA*	Vector for inducible expression of His_6-tagged *scyA*	This study

Table S3. Primers used in this study.

Primer	Nucleotide sequence (5' to 3')
In-frame deletion	
hmuZ-LF	TTGGGATGTGTACTTAGCATGGG
hmuZ-LR	CGCAGTGCCATAGAAAGACT
hmuZ-5O	GGGGACAAGTTTGTACAAAAAAGCAGGCTTGAGAAGTATCAGCAGGCGTG
hmuZ-5I	AGTAGTACAGCCCGCGTCATGCTCGGAAATTTCAGGCTTCATATT
hmuZ-3O	GGGGACCACTTTGTACAAGAAAGCTGGGTCGGTAAAACGCGAAATCACCT
hmuZ-3I	ATGACGCGGGCTGTACTACTGCCTAAGTTAGCCTCGTTGATAGA
hmuZ-SF	TTAACCACCATAGTGATGCTTTCGG
hmuZ-SR	ATTGTTGAGACACTGCGGAC
Complementation	
pHGEN-P*tac*-*hmuZ*-F	GAGAGAATTCCGGATGAAGCCTGAAATTT
pHGEN-P*tac*-*hmuZ*-R	GCCAAGCTTCCGTTAGTGATGGTGATGGTGATGGGCTACCTGCATATCTTCAGG
pHGEN-P*tac*-*degQ*-F	GAAAACGAAATTATCTGTACTTTCAGCC
pHGEN-P*tac*-*degQ*-R	CAGCACCTTCTTGATCTTTGAGC
pHGEN-P*tac*-*hutZ*-F	CACACAGGAGAGAATTCATGGATCAGCAAGTTAAGCAA
pHGEN-P*tac*-*hutZ*-R	CGCCAAAACAGCCAAGCTTTTAGCCATTAGAAATCTTACG
pHGEN-P*tac*-*hugZ*-F	CACACAGGAGAGAATTCATGCTTAATCGTATCATAGAACAC
pHGEN-P*tac*-*hugZ*-R	GCCAAAACAGCCAAGCTTTTATTTCTTGTGAGCG
pHGEN-P*tac*-*c*HOA-F	CAATTTCACACAGGAGAGAATTCATGAAGATCGACCCG
pHGEN-P*tac*-*c*HOA-R	CCAAAACAGCCAAGCTTTCACTCCGCCGGGGGTTTCAGC
Site-directed mutagenesis	
$HmuZ^{(T29V)}$-F	GAAACCTTTAAACAACAACGTAAAGTCTTACAACTCGCCACCGTCG
$HmuZ^{(T29V)}$-R	AATTTCAGGTAACAGTTTGTCCCTTAAACGCTGTTCTTGCTC
$HmuZ^{(R94A)}$-F	GCAAAATCCGTATTCGCCCGCAAAGCTTTAACCTTTGATGCCATGG
$HmuZ^{(R94A)}$-R	GCTTCAGCTTCATCCTCCACCAGCATGACAGACACTTTAGTC
$HmuZ^{(H172A)}$-F	GGAAGCGCGATGGTGCTCACGGCACGCCAAAAACGCC
$HmuZ^{(H172A)}$-R	AGTTAACATCAAGCAGTTCGCTGCCTGTCAAACTAAAGGC
$HmuZ^{(L132A/D134L)}$-F	CGGATAATTTGGCGGCGGCTACGCTGTTTAACCTGTACAAG
$HmuZ^{(L132A/D134L)}$-R	CATGTCGCCAAAACGTTTAGATAACAGGCTGACTCCTTTGC

Supporting Information References

1. Wang J, Guo Q, Li X, Wang X, Liu L. The Arabidopsis locus AT3G03890 encodes a dimeric β-barrel protein implicated in heme degradation. Biochem J. 2020;477 (24): 4785–4796.

2. Emborg TJ, Walker JM, Noh B, Vierstra RD. Multiple heme oxygenase family members contribute to the biosynthesis of the phytochrome chromophore in Arabidopsis. Plant Physiol. 2006;140(3):856–68.

3. Apitz J, Nishimura K, Schmied J, Wolf A, Hedtke B, van Wijk KJ, et al. Posttranslational Control of ALA Synthesis Includes GluTR Degradation by Clp Protease and Stabilization by GluTR-Binding Protein. Plant Physiol. 2016;170(4):2040–51.

4. Ahmed FH, Carr PD, Lee BM, Afriat-Jurnou L, Mohamed AE, Hong NS, et al. Sequence-Structure-Function Classification of a Catalytically Diverse Oxidoreductase Superfamily in Mycobacteria. J Mol Biol. 2015;427(22):3554–71.

5. Li S, Isiorho EA, Owens VL, Donnan PH, Odili CL, Mansoorabadi SO. A noncanonical heme oxygenase specific for the degradation of c-type heme. J Biol Chem. 2021;296:100666.

6. Jin M, Jiang Y, Sun L, Yin J, Fu H, Wu G, et al. Unique organizational and functional features of the cytochrome c maturation system in Shewanella oneidensis. PLoS One. 2013;8(9):e75610.

7. Meng Q, Sun Y, Gao H. Cytochromes c Constitute a Layer of Protection against Nitric Oxide but Not Nitrite. Appl Environ Microbiol. 2018;84(17).
